# Epithelial-mesenchymal transition-related genes in coronary artery disease

**DOI:** 10.1515/med-2022-0476

**Published:** 2022-04-22

**Authors:** Xiang Xu, Renchao Zou, Xiaoyong Liu, Jia Liu, Qianqian Su

**Affiliations:** Department of Cardiology, The Second Affiliated Hospital of Kunming Medical University, Kunming City, Yunnan Province, China; Department of Hepatobiliary Surgery, The Second Affiliated Hospital of Kunming Medical University, Kunming City, Yunnan Province, China; Department of Laboratory Animal Science, Kunming Medical University, Kunming City, Yunnan Province, 650500, China

**Keywords:** coronary artery disease, epithelial-mesenchymal transition, qPCR, lncRNA, potential biomarker

## Abstract

Epithelial-mesenchymal transition (EMT) is critical in the development of coronary artery disease (CAD). However, landscapes of EMT-related genes have not been fully established in CAD. We identified the differentially expressed mRNAs and lncRNAs (DElncRNAs) from the Gene Expression Omnibus database. Pearson’s correlation analysis, the least absolute shrinkage and selection operator regression, and support vector machine reverse feature elimination algorithms were used to screen EMT-related lncRNAs. The cis–trans regulatory networks were constructed based on EMT-related lncRNAs. Quantitative real-time polymerase chain reaction was performed to validate the expression of EMT-related genes in a cohort of six patients with CAD and six healthy controls. We further estimated the infiltration of the immune cells in CAD patients with five algorithms, and the correlation between EMT-related genes and infiltrating immune cells was analyzed. We identified eight EMT-related lncRNAs in CAD. The area under curve value was greater than 0.95. The immune analysis revealed significant CD8 T cells, monocytes, and NK cells in CAD and found that EMT-related lncRNAs were correlated with these immune cell subsets. Moreover, SNAI2, an EMT-TF gene, was found in the trans-regulatory network of EMT-related lncRNAs. Further, we found SNAI2 as a biomarker for the diagnosis of CAD but it also had a close correlation with immune cell subsets in CAD. Eight EMT-related lncRNAs and SNAI2 have important significance in the diagnosis of CAD patients.

## 1 Introduction

Coronary artery disease (CAD) is a common public health problem, mainly occurring in people over 45 years of age. CAD is now the leading cause of death in the United States, accounting for one in six deaths alone [1]. The American Heart Association has said that cardiovascular disease causes more than 17.3 million deaths a year; by 2030, the number of deaths will exceed 23.6 million [2]. CAD is the most common type of cardiovascular disease. Its pathogenesis is due to coronary artery atherosclerotic lesions resulting in the narrowing of vascular lumen causing obstruction and reduced blood supply to the myocardium, hypoxia or necrosis, and eventually leading to heart failure [3,4]. However, the pathogenesis of CAD is complex, and there are no apparent symptoms in the early stage. The results of the myocardial enzyme spectrum can be negative, which is only manifested by abnormal ST-T segment changes in the exercise plate electrocardiogram. Although coronary angiography is the gold standard for diagnosing CAD, its high cost and technical requirements, reliance on specific equipment, and potential risk of radionuclide radiation have limited its use. Meanwhile, it is neither practical nor ethical to perform invasive coronary angiography on low-risk patients [5]. The cost of blood biomarker detection is low and easy to promote [6]. Therefore, it is vital to search for more potential biomarkers for the diagnosis and treatment of CAD based on blood sequencing data.

Epithelial-mesenchymal transition (EMT) is a biological process in which epithelial cells are transformed into cells with a mesenchymal phenotype through a specific procedure. During this process, endothelial cells gradually lose their morphology and function and acquire the phenotypic characteristics of mesenchymal cells such as proliferation, migration, and collagen synthesis [7]. Recent studies have demonstrated that EMT plays a pivotal physiological and pathological role in the development and structural remodeling of the myocardium, blood vessels, and valves, suggesting that EMT may be a worthwhile target for preventing and treating cardiovascular diseases. For example, endocardial EMT generates valvular cells necessary for heart valve formation and complete septal formation [8]. Epicardial EMT also generates cardiac fibroblasts, vascular smooth muscle cells, and surrounding cardiomyocytes necessary for cardiac muscle growth and coronary angiogenesis [9]. In CAD, endothelial cells participate in the formation of fibroblasts through the mesenchymal transformation of epithelial cells and promote cardiac fibrosis [10]. In addition, the EMT of endothelial cells plays a crucial role in the process of atherogenesis [11]. These studies suggest that EMT genes have an important significance in the field of cardiovascular disease.

Long-coding RNAs (lncRNAs) are noncoding RNAs with a length of more than 200 nucleotides. They have a wide range of functional activities, including RNA decay, genetic regulation of gene expression, RNA splicing, microRNA (miRNA) regulation, and protein folding [12]. LncRNAs also play an essential role in forming atherosclerosis, CAD, and heart failure [13]. For example, several genome-wide association studies have found that some single nucleotide polymorphisms located in lncRNA-ANRIL are closely related to the susceptibility to atherosclerosis and are also the sites with the most potent genetic susceptibility in CAD [14,15]. However, among all the identified lncRNAs, only a few have been verified as being involved in the regulation of EMT. For example, metastasis-associated lung adenocarcinoma transcript 1 (MALAT1) is a lncRNA competing with miRNAs, directly interacts with oncogenes and proteins, and is involved in the activation of Wnt/β-catenin, PI3K/Akt/mTOR – these are typical EMT-related signal pathways [16,17]. A novel study indicated that MALAT1/miRNA-203/Wnt5a axis was a potential regulate mechanism for CAD [18]. Currently, the number of EMT-related lncRNAs in CAD research is small. How EMT-related lncRNAs regulate the formation and progression of CAD remains unclear.

Therefore, based on the previous research, we constructed two machine learning algorithms: least absolute shrinkage and selection operator (LASSO) regression algorithm and support vector machine reverse feature elimination (SVM-RFE) algorithm to screen out EMT-related diagnostic lncRNAs in CAD patients. Meanwhile, we constructed cis–trans regulatory networks based on EMT-related lncRNAs and explored the potential EMT gene of related molecules in the cis–trans network and the target drugs and structures. We also investigated the correlation between EMT-related diagnostic signatures and immune cell subsets by immune analysis. Through bioinformatics methods, an in-depth excavation of the EMT genes as having a promoting role in coronary atherosclerosis and the potential signal pathways and molecular mechanisms for the prevention and treatment of CAD, can provide a new train of thought and targets.

## 2 Methods and materials

### 2.1 Data collection

For our study, we downloaded the microarray gene expression profiling data of CAD from the Gene Expression Omnibus (GEO; https://www.ncbi.nlm.nih.gov/geo/) database with accession number GSE113079 [19]. The platform for GSE113079 was GPL20115, Agilent-067406 Human CBC lncRNA + mRNA microarray V4.0 (Probe name version), which contained peripheral blood mononuclear cells of 93 CAD patients and 48 healthy controls. Two hundred EMT-related genes were obtained from the Molecular Signatures Database (MsigDB, http://www.broad.mit.edu/gsea/msigdb/). Besides, 1,639 genes related to TFs were acquired from the database of The Human Transcription Factors (TFBS, http://tfbsdb.systemsbiology.net/).

### 2.2 Differentially expressed analysis

The limma package in R was used to identify the differentially expressed lncRNAs (DElncRNAs) and DE genes (DEGs) between CAD and normal samples (Tables S1 and S2). The lncRNAs/mRNAs met the selection standards of |(Fold change (FC))| >1.5 and false discovery rate (FDR) <0.01 and were considered as DElncRNAs/DEGs for further study.

### 2.3 Correlation analysis

By mating the listed 200 EMT-related genes in the MsigDB database, DE EMT genes for CAD were identified. Then, Pearson’s correlation analysis was operated between the harvested DE-EMTs and DElncRNAs expression data in samples to identify the EMT-related lncRNAs according to the correlation coefficient and *P* values (|Cor| >0.8 and *P* < 0.05) (Table S3).

### 2.4 Diagnostic value of EMT-lncRNAs and SNAI2

To explore the diagnostic ability of EMT-lncRNAs mentioned above, receiver operating characteristic (ROC) analysis was first performed using the R package pROC, and the EMT-related lncRNAs with area under curve (AUC) >0.95 were screened for further study. After filtration of EMT-related lncRNAs, candidate diagnostic lncRNAs for CAD were selected via an integrated analysis of two algorithms consisting of LASSO and SVM-RFE. Logistic regression was performed on diagnostic lncRNAs and the SNAI2 gene, respectively, to construct a logistic regression diagnostic model, and the bias residual diagram was drawn (Figure S1a and b). Five-fold cross validation was used to evaluate the performance of the diagnostic signature. Moreover, the diagnostic value of EMT-related lncRNAs and SNAI2 was assessed by ROC curve analysis using the pROC package in the R language.

### 2.5 EMT-related lncRNAs categorization

Based on modifications of the previous classification [20], we classified lncRNAs according to their gene positions related to the most proximal protein-coding genes. First, based on whether they intersect a protein-coding gene, the lncRNA genes were regarded as intergenic and genic. Furthermore, intergenic lncRNAs were categorized into two groups depending on whether they were their transcribed from the same or opposite strands: convergent (IC) and divergent (ID). Genic lncRNAs were separated into genic exonic (genic exonic same strand [GES] and genic exonic antisense [GEAS]), genic intronic (genic intronic same strand [GIS] and genic intronic antisense [GIAS]), or overlapping (genic overlapping same strand [GOS] and genic overlapping antisense [GOAS]) based on whether they overlapped with the exons or introns of a protein-coding gene.

### 2.6 The regulatory mechanisms of diagnostic EMT-lncRNAs

It is reported that lncRNAs regulated transcription of the genes nearby by acting in cis- and tans-manners. For the cis-regulation manner, we first selected the genes located on the same chromosome within a 300 kb region upstream or downstream of the lncRNAs. Subsequently, the Pearson analysis method was performed to analyze the correlation between the harvested lncRNAs and their corresponding genes under the selection criteria of |Cor| >0.3 and *P* < 0.05.

For trans prediction, we focused on the fact that lncRNAs might regulate the expression levels of TFs by the trans manner. After selecting the genes correlated with lncRNAs by the Pearson method (|Cor| >0.8 and *P* < 0.05), we further overlapped these genes with identified DEGs and TFs to obtain trans-regulated genes. A lncRNA–mRNA network that included EMT-lncRNAs, cis- and trans-regulated genes was constructed and visualized by the Cytoscape software.

### 2.7 Functional enrichment analysis

To explore the latent biological functions and pathways, gene ontology (GO) annotation and kyoto encyclopedia of genes and genomes (KEGG) pathway analyses were employed on the DE-EMTs, cis-regulated genes, trans-regulated genes, and SNAI2-regulated genes of CAD, respectively. The optional pathways related to CAD were predicted by the Comparative Toxicogenomics Database (CTD, http://ctdbase.org). Genes related to CAD were predicted by the DisGeNET database (https://www.disgenet.org/home/). The KEGG pathways both in the CAD database and KEGG analysis were introduced into the lncRNA–mRNA network to establish a lncRNA–mRNA-pathway network for CAD.

### 2.8 Immunity analysis and its correlation with key genes

We used cell type identification by estimating relative subsets of RNA transcripts algorithm (CIBERSORT) [21] for immune infiltration. R script downloaded from CIBERSORT website (https://cibersort.stanford.edu/). After obtaining the immune cell expression matrix according to the instructions on the CIBERSORT website, we used the “ggplot2” software package to create a cumulative histogram that visually showed the proportion of 22 immune cell infiltrates in CAD patients. We also used the “vioplot” package to draw violin plots showing differences in the expression of 22 infiltrating immune cells. We used the “corrplot” software package in R software to calculate Pearson correlation coefficients among immune cells, and displayed the results by using a correlation heat map. Pearson correlation coefficients and *p* value between identified key genes and infiltrating immune cells were calculated by “cor” and “Hmisc” software packages and then visualized by the “ggcorrplot” software package. In addition, single-sample gene set enrichment analysis (ssGSEA) [22], microenvironment cell population (MCP)-counter algorithm [23], EPIC [24], and QuanTIseq [25] algorithms were also used to compare and assess cellular components between the high SNAI2 and low SNAI2 gene expression groups. The differences in the immune response under different algorithms were uncovered using a Heatmap.

### 2.9 The drug–gene prediction

The genes cis- and trans-regulated by diagnostic lncRNAs were supposed to be the promising drug targets for searching drugs through the Drug–Gene Interaction database (DGIdb, https://dgidb.genome.wustl.edu/) that contained the DGI information of several databases [26]. The drug–gene network was visualized by the Cytoscape tool.

### 2.10 Study population

A total of six CAD patients and six healthy controls were recruited as a validation cohort in the Second Affiliated Hospital of Kunming Medical University, Kunming, China. This study is in accordance with the Declaration of Helsinki and was approved by the Ethics Committee of the Second Affiliated Hospital of Kunming Medical University (No. PJ-2022-14). All subjects were aged >18 years. The diagnosis of CAD is based on the European Society of Cardiology criteria [27], which specifies at least one vascular lesion with a narrowing of lumen diameter greater than 50% using coronary angiography. Healthy subjects had no history of chronic disease and CAD. All participants signed informed consent after receiving a complete study explanation.

### 2.11 RNA extraction and quantitative real-time polymerase chain reaction (qPCR)

The expression of target genes was detected by qPCR to validate our microarray data. Briefly, EDTA vacuum anticoagulant vessels were used to collect 4–8 mL of peripheral blood from patients with CAD and healthy controls in the morning. The total RNA was extracted from peripheral blood cells of patients with CAD using TRIzol reagent (Invitrogen) and reverse-transcribed into cDNA (SweScript RT I Frist Strand cDNA Synthesis Kit, Servicebio, Wuhan, China). qPCR was performed using 2× Universal Blue SYBR Green qPCR Master Mix (Servicebio, Wuhan, China) following the manufacturer’s instructions. Glyceraldehyde-3-phosphate dehydrogenase (GAPDH) was measured as an internal control. The reaction conditions were 95°C for 1 min, 40 cycles at 95°C for 20 s, 55°C for 20 s, and 72°C for 30 s. The melting curve was analyzed for each sample. The average value in each duplicate was used to calculate the relative amount of target genes using the 2^−ΔΔCt^ method. All primers were synthesized by TSINGKE (TSINGKE Biotechnology CO., Ltd, Beijing, China). The primer sequences of the tested genes are shown in Table 1. The mRNA levels of lncRNAs between CAD and normal groups were compared using *t* test (*P* < 0.05) using *R*.

**Table 1:** Primers used for qPCR analysis of eight EMT-related lncRNAs and SNAI2

Gene names	Position	Primer sequence
CTD-2089N3.3	Forward	GACGACCACCACGCAGGAG
	Reverse	GGAGAGGGGGAACAAGGCT
AC113167.2	Forward	TTGGCTTCCTTACCTTGAGT
	Reverse	CCGATTATCCTTTTTCTTCC
LINC02747	Forward	TGTGTGCGTCCTCCCTAAA
	Reverse	AGCAGAGACAGAGCCGGTT
RP11-1152H15.1	Forward	GGTCCACACTGCTTTTATG
	Reverse	CTCTAGTCCTTCGGCTCAA
LINC02833	Forward	CAGACAAAATCAAAATGGAG
	Reverse	TAAGGTGGGAGTAAGGAAAC
AC109460.4	Forward	GACTGTCCCTAATTTCCCCT
	Reverse	TGTCTGACTCGTTCTCCTCA
LINC01775	Forward	AGACAGGAGGGCTGGGGT
	Reverse	GGAGGCTGAGGCAGGAGA
RP11-103H7.3	Forward	CAGTTTCTGCCCACAATGA
	Reverse	GTGTAACCTCTCCAGCCCT
SNAI2	Forward	AAACTACAGCGAACTGGACA
	Reverse	ATAGAGATACGGGGAAATAA
H-GAPDH	Forward	CCCATCACCATCTTCCAGG
	Reverse	CATCACGCCACAGTTTCCC

### 2.12 Statistical analysis

The subcellular localization of diagnostic EMT-related lncRNAs was predicted by the LncLocator online tool [28]. The clusterProfiler package in R was utilized to perform GO and KEGG analyses. The statistical analyses were performed using R (R Foundation for Statistical Computing, Vienna, Austria, version 4.0.3) (http://www.R-project.org/) and GraphPad Prism (Version 8 GraphPad Software, La Jolla, CA, USA). *P* value <0.05 was considered as statistically significant.


**Ethics statement:** The study was carried out in accordance with the recommendations of the Ethics Committee of the Second Affiliated Hospital of Kunming Medical University (No. PJ-2022-14) and was approved by said committee. All participants signed informed consent after receiving a complete study explanation.
**Informed consent:** A preprint has previously been published and PREPRINT (Version 2) available at Research Square, and DOI is https://doi.org/10.21203/rs.3.rs-940366/v2.

## 3 Results

### 3.1 Identification of EMT-related genes

**Figure 1:**
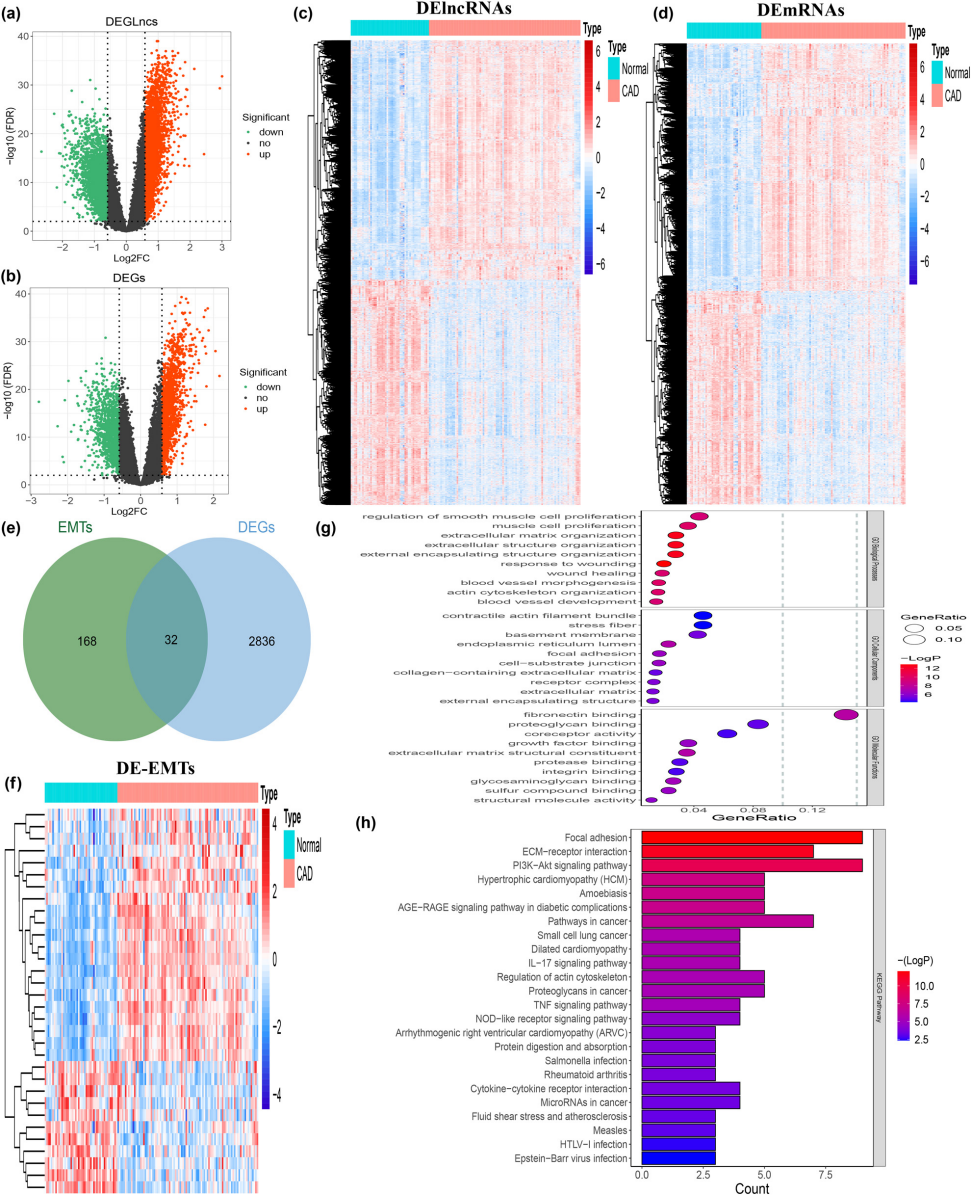
Identification of DE-EMTs in CAD. (a) Volcano plot of lncRNAs expression between CAD and normal groups. (b) Volcano plot of mRNAs expression between CAD and normal groups. (c) Heatmap of DElncRNAs between CAD and normal groups. (d) Heatmap of DEGs between CAD and normal groups. (e) Venn diagram was used for the intersection of DEGs and EMT genes. (f) Heatmap of DE-EMTs between CAD and normal groups. (g) GO enrichment analysis of DE-EMTs: the larger the bubble and longer columns represent the more genes enriched in this function, the deeper the color of the bubble and bars, the smaller the *P* value. (h) KEGG enrichment analysis of DE-EMTs.

We performed the differentially expressed analysis on the GSE113079 data set. As shown in Figure 1a, 5,955 DElncRNAs were identified between CAD and normal samples under |(FC)| >1.5 and FDR <0.01 with 3,067 were upregulated and 2,888 downregulated. Meanwhile, we screened 2,868 DEGs between the two groups, including 1,540 upregulated and 1,328 downregulated DEGs (Figure 1c). The expressed levels of DElncRNAs and DEGs were shown in the heatmap plot and displayed in Figure 1b and d, respectively. The 32 DEGs related to EMT were generated by overlapping 200 EMT genes in the MsigDB database and preselected DE-EMTs, in which 21 were upregulated and 11 were downregulated (Figure 1e and f).

To further reveal the potential mechanism of the role of the above 32 DE-EMT in CAD, we performed functional enrichment analysis by Metascape software. A total of 262 biological process (BP) terms, 26 molecular function (MF) terms, and 26 cellular component terms were enriched in the GO system (Figure 1g). We mainly focused on the enrichment results of GO-BP categories. The “response to wounding” was the most significantly enriched term, speculating that these genes may be involved in the regulation of the response after coronary artery damage. Next, “extracellular matrix organization” and “extracellular structure organization” were significantly enriched, suggesting that these genes may be associated with the acquisition of a mesenchymal phenotype by epithelial cells after EMT. The enrichment of terms related to cell adhesion (“regulation of cell adhesion,” “cell-matrix adhesion,” “positive regulation of cell adhesion,” “cell-substrate adhesion,” “regulation of cell-substrate adhesion,” etc.) and cytoskeletal (“actin cytoskeleton organization,” “regulation of actin cytoskeleton organization,” “regulation of cytoskeleton organization,” “positive regulation of cytoskeleton organization,” etc.) regulation and extracellular matrix disassembly could also be confirming the possibility of this speculation. Surprisingly, we found that terms related to the dynamic developmental processes of the vasculature (“blood vessel morphogenesis,” “blood vessel development,” “vasculature development,” “angiogenesis,” etc.) and cardio (“heart development,” “semi-lunar valve development,” “heart morphogenesis,” “heart valve morphogenesis,” “heart valve development,” etc.) were also significantly enriched, implying that DE-EMT may be involved in the process of CAD onset and development. Besides, terms related to the regulation of biological processes such as activation and differentiation of B cells, lymphocytes, myeloid leukocytes, and terms related to immune response (“negative regulation of immune system process,” “cell activation involved in immune response,” “humoral immune response,” etc.) were also closely related to these genes. The enrichment results on the GO system are available in Table S4. KEGG pathway enrichment showed that 32 DE-EMT were involved in a total of 24 pathways (Figure 1h; Table S5). Among them, “PI3K-Akt signaling pathway” and “focal adhesion” were the two most significantly enriched pathways. Moreover, these genes were also linked to multiple CAD-related cardiac disease pathways, such as “IL-17 signaling pathway,” “PI3K-Akt signaling pathway,” and “Fluid shear stress and atherosclerosis.” This evidence further suggested that the 32 CAD-related DE-EMT genes may play a role in the CAD process through a certain EMT mechanism.

### 3.2 Identification of EMT-related lncRNAs

**Figure 2:**
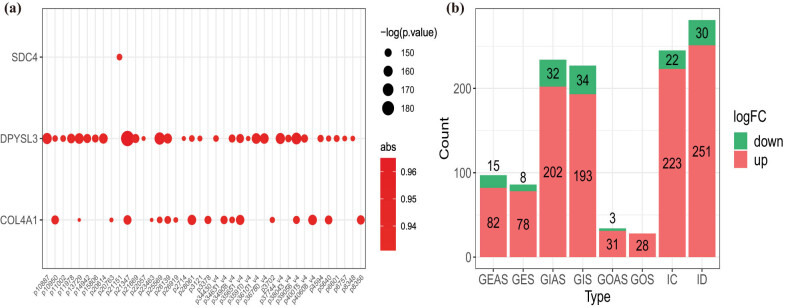
Identification of EMT-related lncRNAs in CAD. (a) Correlation analyses for EMT-related lncRNAs. (b) Classification bar diagram of EMT-related lncRNAs.

Next, we screened the lncRNAs related to EMT in CAD with correlation analysis. According to the screening criteria, 1,141 EMT-related lncRNAs were identified. The Top50 EMT-lncRNAs are illustrated in Figure 2a. Genic lncRNAs stand for the largest category (57.2%) of EMT-related lncRNAs (GEAS = 7.8% and GES = 7%, GIAS = 19% and GIS = 18.4%, GOAS = 2.8% and GOS = 2.3%), following intergenic lncRNAs were 42.7% (IC = 19.9% and ID = 22.8%) (Figure 2b).

### 3.3 Construction of an EMT-related lncRNAs diagnostic signature for CAD

**Figure 3:**
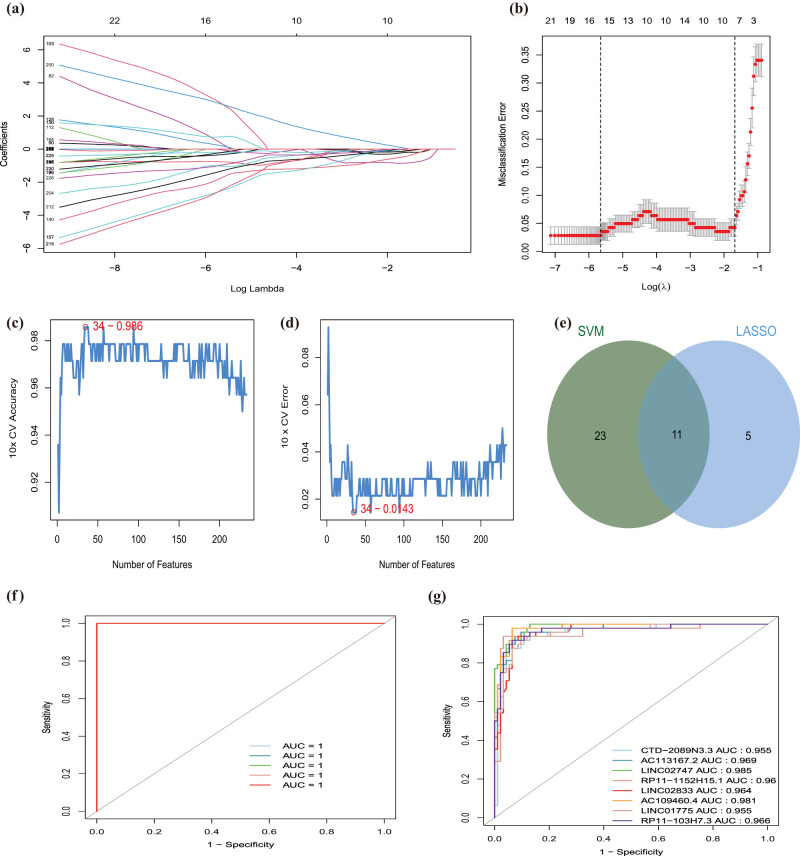
EMT-related lncRNAs diagnostic signature for CAD. (a) LASSO coefficient profiles of the 16 EMT-related lncRNAs selected by the optimal lambda. (b) The diagnostic signature selection of optimal parameter (lambda) in LASSO model. (c and d) Results of SVM-RFE algorithms: The point highlighted indicates the lowest error rate, and the corresponding EMT-related lncRNAs at this point are the best signature selected by SVM-RFE. (e) Venn diagram of overlap EMT-related lncRNAs selected by LASSO and SVM-RFE algorithms. (f) The ROC curve of predicted outcomes of eight EMT-related lncRNAs diagnostic signature by a logistic regression model. (g) ROC analysis results for eight EMT-related lncRNAs in CAD.

To further detect the diagnostic ability of these EMT-related lncRNAs, the AUC value of each EMT-related lncRNAs was analyzed. Two hundred twenty-three EMT-related lncRNAs were screened with an AUC value above 0.95 (Table S6). LASSO regression analysis and SVM-RFE algorithm were used to identify the optimal diagnostic lncRNAs in the GSE113079 data set and establish the risk signature for CAD. Sixteen EMT-related lncRNAs were screened via the LASSO analysis, which intersected with 34 EMT-related lncRNAs obtained from the SVM-RFE algorithm to identify 11 diagnostic lncRNAs for CAD (Table S7, Figure 3a–e). After annotating the diagnostic lncRNAs using the Rsubread package in R, we obtained eight lncRNAs that were used to construct a diagnostic signature for CAD (Table S8), showing accuracy and specificity for the diagnosis of CAD (AUC = 1) (Figure 3f). Besides, the AUC value of each diagnostic lncRNAs was greater than 0.95, which exhibited a better ability to distinguish CAD patients from normal individuals (Figure 3g). Subcellular localization of each lncRNA determines the regulatory models. To investigate the subcellular localization of the diagnostic lncRNAs, we assessed LncLocator online platforms. We uncovered that these diagnostic lncRNAs were mainly located in the cytosol and cytoplasm (Figure S2)

### 3.4 Establishment of cis- and trans-regulatory network

**Figure 4:**
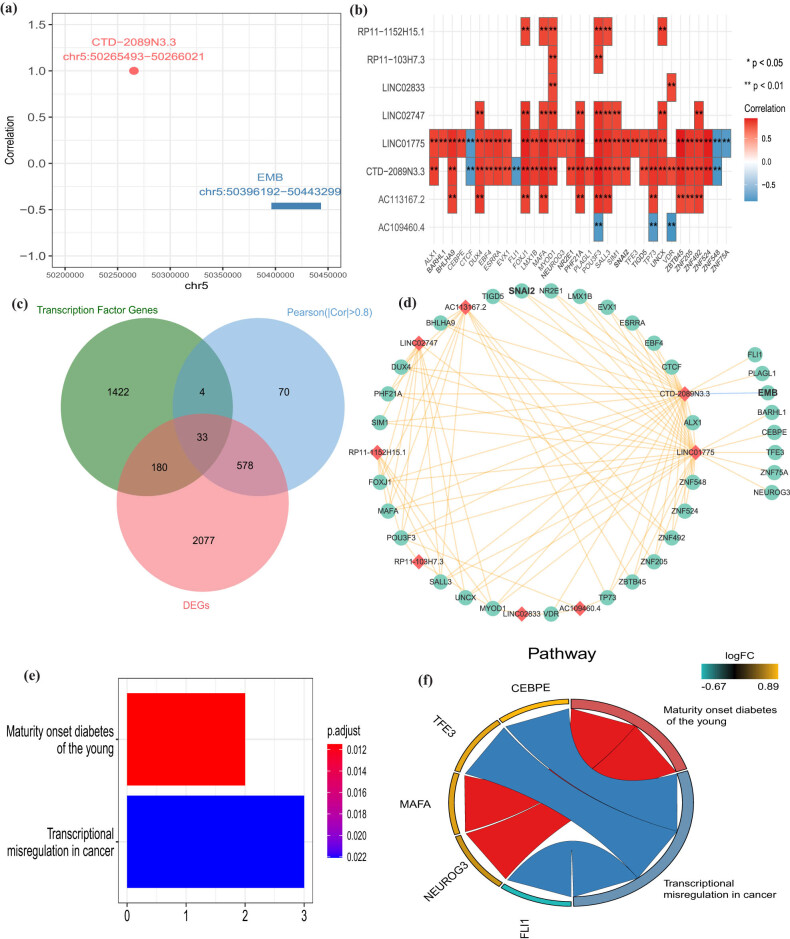
The cis and trans network of the EMT-related lncRNAs. (a) Cis-regulation gene of lncRNA CTD-2089N3.3 in the chromosome. The X-axis represents lncRNA position in chromosome and the Y-axis represents correlation coefficient of lncRNA and the potential “cis” gene. The red line represents the genome width of lncRNA and blue point represents the position of potential “cis” gene. (b) Heatmap of the correlations between eight EMT-related lncRNAs and trans-regulated genes. (c) Venn diagram of 33 “trans” genes regulated by eight EMT-related lncRNAs. (d) The cis and trans network of the eight EMT-related lncRNAs (red) in CAD and their target mRNAs (green). (e and f) KEGG enrichment analysis of 34 cis and trans genes regulated by EMT-related lncRNAs.

Previous studies indicated that lncRNAs regulated gene expression via local (cis) and long-distance (trans) mechanisms [29]. In this study, we identified seven diagnostic lncRNAs regulated their nearby genes via the cis-regulatory manner, except RP11-103H7.3 (Table 2). Among them, only CTD-2089N3.3 were significantly correlated with their corresponding gene EMB via Pearson analysis under |Cor| >0.3 and *P* value <0.05 (Figure 4a). Based on the median expression level of EMB, we divided the CAD patients in the GSE113079 data set into the high-expressed and low-expressed EMB groups. GSEA result suggested that several metabolic- and tumor-related pathways were associated with high-expressed EMB groups, including “fatty acid metabolism,” “pyrimidine metabolism,” “mTOR signaling pathway,” and “TGF BETA signaling pathway.” In contrast, “calcium signaling pathway,” “complement, and coagulation cascades,” “neuroactive ligand–receptor interaction,” and “olfactory transduction” were involved in the low-expressed EMB group (Figure S3).

**Table 2:** Seven EMT-related diagnostic lncRNAs regulated their nearby genes via the cis-regulatory manner

Gene	lncRNA	Symbol	Cor	*P* value	Chromsome	Distance
EMB	p22710	CTD-2089N3.3	−0.46194075	8.12 × 10^−9^	chr5	130,172
MEF2C	p40724_v4	AC113167.2	0.156713247	0.063484128	chr5	−294,003
MYEOV	p2383	LINC02747	0.23717925	0.004628671	chr11	0
KCNK10	p4630	RP11-1152H15.1	−0.251517089	0.002625124	chr14	0
UNC79	p4940	LINC02833	−0.073789859	0.384523053	chr14	0
LAT	p5961	AC109460.4	−0.244589931	0.003466511	chr16	−9,677
LMNB2	p8126	LINC01775	0.05339002	0.529495266	chr19	−1,974

By combining 685 genes correlated with lncRNAs with DEGs and identified TFs, 33 genes were identified to be regulated by diagnostic lncRNAs via trans manner, in which SNAI2 was founded to be a DE-EMT (Figure 4b and c). Then, a lncRNA–mRNA regulatory network was constructed that contained diagnostic lncRNAs, cis- and trans-regulated genes, which consisted of 42 nodes and 93 edges (Figure 4d). These genes in the regulatory network were mainly involved in nervous development and vitamin metabolism by GO analysis (Figure S4a). Combining the pathways related to these genes identified with the KEGG analysis and related to CAD development in the CTD database, “maturity onset diabetes of the young” and “transcriptional misregulation in cancer” were discovered (Figure 4e and f, Table 3). Hence, these two KEGG pathways were introduced into the lncRNA–mRNA regulatory network to establish a lncRNA–mRNA pathway network for CAD that included 46 nodes and 98 edges (Figure S4b).

**Table 3:** Combining the pathways related to potential “cis” and “trans” genes identified with the KEGG analysis and related to CAD development in the CTD database

# Input	DiseaseName	PathwayName	PathwayID	Inference GeneSymbol
Coronary heart disease	Coronary disease	Maturity onset diabetes of the young	KEGG:hsa0495 0	HNF1A
Coronary heart disease	Coronary disease	Transcriptional misregulation in cancer	KEGG:hsa0520 2	MMP3
Coronary heart disease	Coronary disease	Transcriptional misregulation in cancer	KEGG:hsa0520 2	PLAU

### 3.5 Prediction of regulatory genes of SNAI2

**Figure 5:**
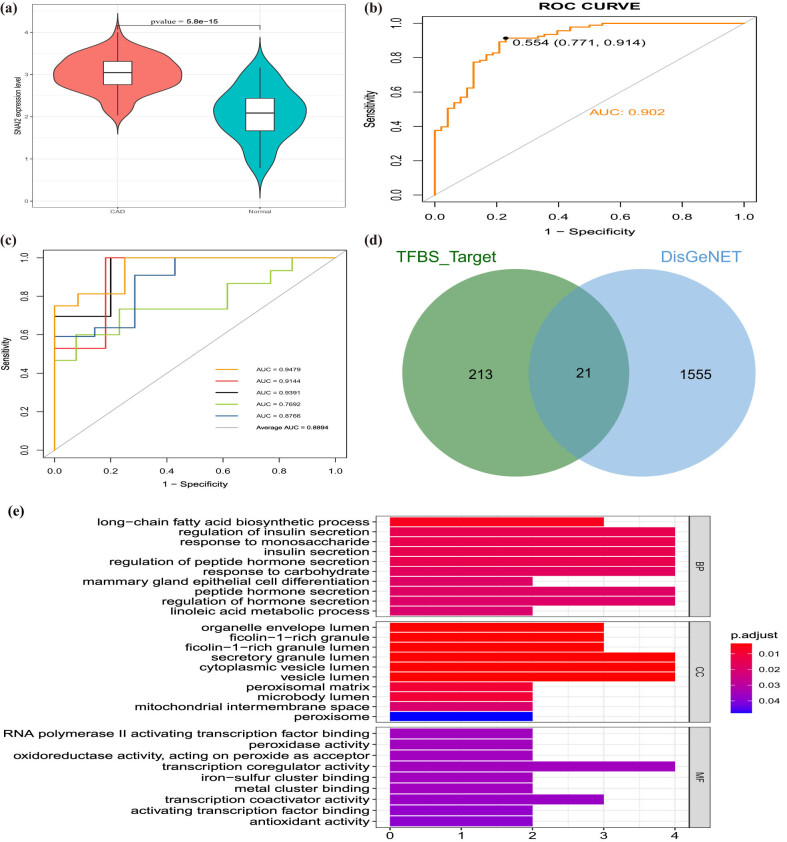
Clinical performance of SNAI2 in CAD. (a) SNAI2 is highly expressed in CAD groups and low in normal groups. (b) ROC analysis results for SNAI2 in CAD. (c) The ROC curve of predicted outcomes of SNAI2 diagnostic signature by a logistic regression model. (d) Venn diagram of 21 CAD-related genes regulated by SNAI2. (e) GO enrichment analysis of 21 CAD-related genes regulated by SNAI2.

Based on the above results, we uncovered that SNAI2 was found to be a DE-EMT and TFs among all genes regulated by diagnostic lncRNAs. In our study, SNAI2 is obviously higher expressed in CAD groups than normal groups (*p* = 5.8 × 10^–15^; Figure 5a). Considering the importance of SNAI2, we also detect the diagnostic ability of SNAI2 in CAD patients. The AUC value of SNAI2 was 0.902 (Figure 5b). In addition, we used five-fold cross validation to evaluate the reliability of the SNAI2 gene. First, we randomly divided the samples into five parts, of which four parts were used as training sets to build the logistic regression model, and the rest were used to verify the model. This process was then repeated five times to reduce errors and improve the sensitivity of the model. The AUC values of the five models were 0.9479, 0.9144, 0.9391, 0.7692, and 0.8766, respectively, indicating that the models had good explanatory power (Figure 5c). Besides, GSEA was performed to investigate the latent biological functions. “Calcium signaling pathway,” “linoleic acid metabolism,” “neuroactive ligand receptor interaction,” and “olfactory transduction” were mainly associated with the high-expressed SNAI2 group. “RNA degradation,” “splicesome,” “fatty acid metabolism,” and “histone metabolism” were involved in the low-expressed SNAI2 group (Figure S5). Moreover, we overlapped 234 genes regulated by SNAI2 acquired from the TFBS database and 1,576 genes related to CAD acquired from the DisGeNET database to obtain 21 genes regulated by SNAI2 for CAD (Figure 5d). Functional enrichment analysis determined that the harvested 21 genes were concerted on the “insulin secretion,” “peptide hormone secretion,” and “long-chain fatty acid biosynthetic process” (Figure 5e). No pathways were detected by the KEGG analysis.

### 3.6 Prediction of the target drugs of genes in the cis- and trans-regulatory network

Next, the target drugs of genes regulated by diagnostic lncRNAs were predicted by the DGIdb database. Through DGIdb prediction, a total of 483 drug–gene pairs were identified, and a target-drug network for CAD was constructed, including five genes and 476 drugs. Four hundred fifty-nine drugs interacted with VDR, which might be promising to treat patients with CAD (Figure S6a). The structures of these drugs are illustrated in Figure S6b–i.

### 3.7 Immune analysis of EMT-lncRNAs and SNAI2

**Figure 6:**
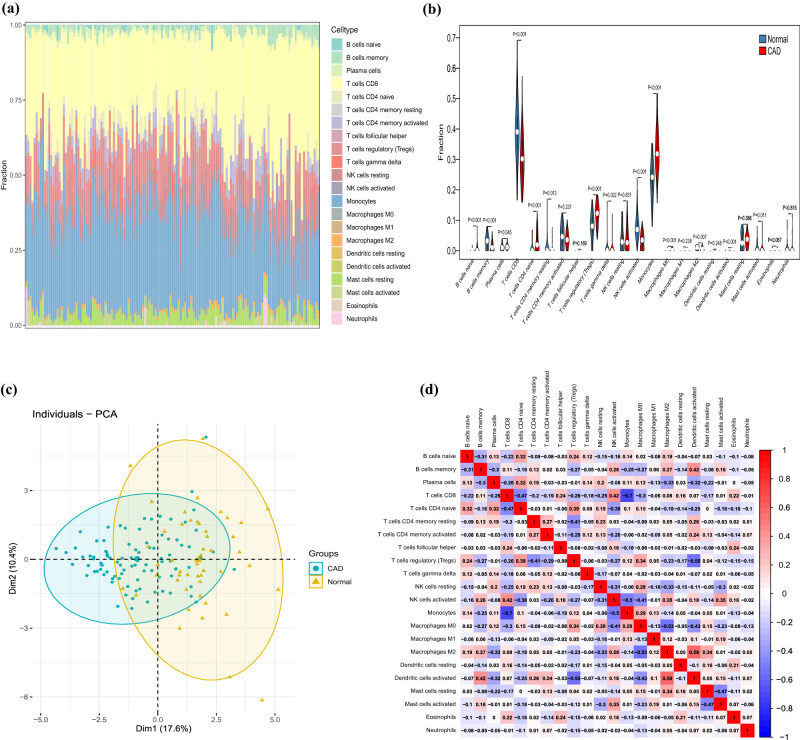
Evaluation and visualization of immune cell infiltration by CIBERSORT algorithm. (a) Immune cell types and ratios of CAD groups. (b) Violin plot comparing immune cell compositions between CAD groups and normal groups. (c) PCA for immune cell compositions between CAD groups and normal groups. (d) Pearson correlation heatmap between infiltrating immune cell subpopulations.

**Figure 7:**
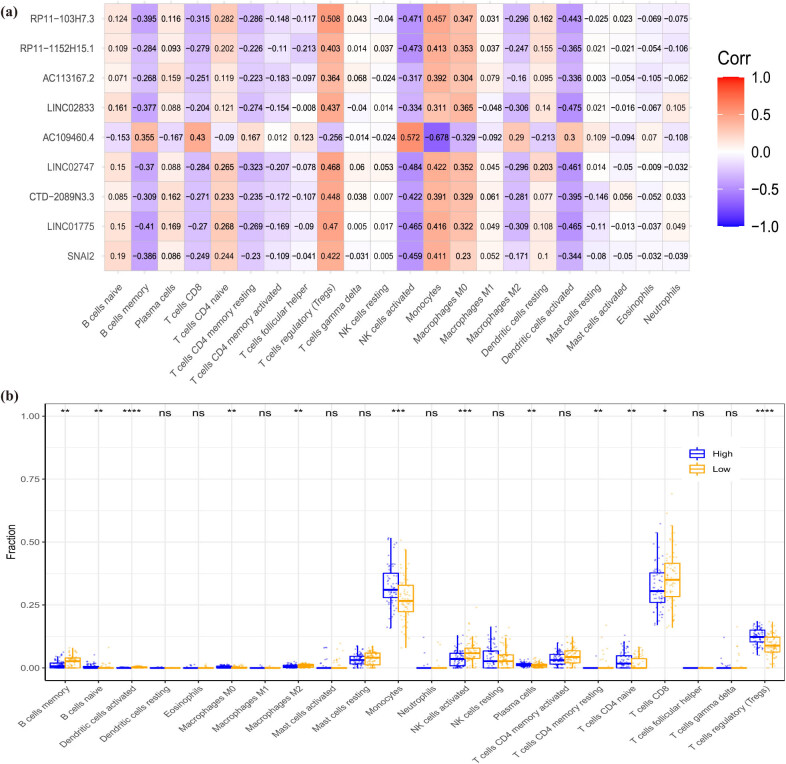
Immune analysis of SNAI2. (a) Pearson correlation heatmap between eight EMT-related diagnostic signatures and infiltrating immune cells. (b) Boxplot comparing immune cell compositions between high SNAI2 expression level groups and low SNAI2 expression level groups.

**Figure 8:**
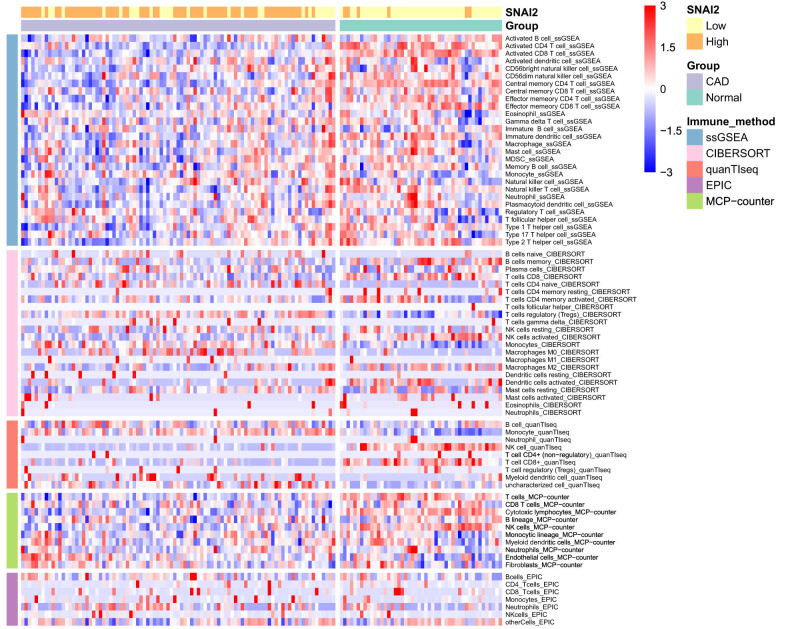
Heatmap for immune cell compositions based on ssGSEA, CIBERSORT, quanTIseq, MCP-counter, and EPIC algorithms among different groups.

Enrichment analysis showed that DE-EMT gene was enriched in inflammatory response-related pathways. Therefore, we evaluated the type and fraction of immune cell infiltration between CAD patients and normal samples in the data set using the CIBERSORT algorithm. The relative proportion of immune cell subtypes is shown in the cumulative histogram (Figure 6a). Our results found an apparent proportion of CD8 T cells, NK cells activated, and monocytes. Moreover, the infiltration of CD8 T cells and NK cell activated were decreased, and the infiltration of monocytes was increased in CAD patients (Figure 6b). By principal component analysis (PCA), immune cell fractions in CAD patients and normal controls showed intergroup bias and individual differences (Figure 6c). In the correlation heatmap (Figure 6d), we found that CD8 T cells were negatively correlated with monocytes and macrophages M0, and positively correlated with NK cells activated. This is consistent with the correlation between seven EMT-related lncRNAs and the immune cells we found, except lncRNA AC109460.4 (Figure 7a). In addition, we also conducted an immune analysis of SNAI2. Then, we divided the samples into high and low groups according to the expression level of SNAI2. It was found that in the high expression level group, the infiltration of monocytes was decreased. In contrast, the infiltration of NK cells activated and CD8 T cells were increased, which was similar to immune cell infiltration in CAD patients (Figure 7b). The heatmap of immune cell compositions based on CIBERSORT, quanTIseq, ssGSEA, MCP-counter, and EPIC algorithms are shown in Figure 8. It was found that CD8 T cells, monocytes, and NK cells activated had similar immune cell infiltration trends in the CAD and SNAI2 gene high expression groups.

### 3.8 Validation of EMT-lncRNAs and SNAI2 expression

**Figure 9:**
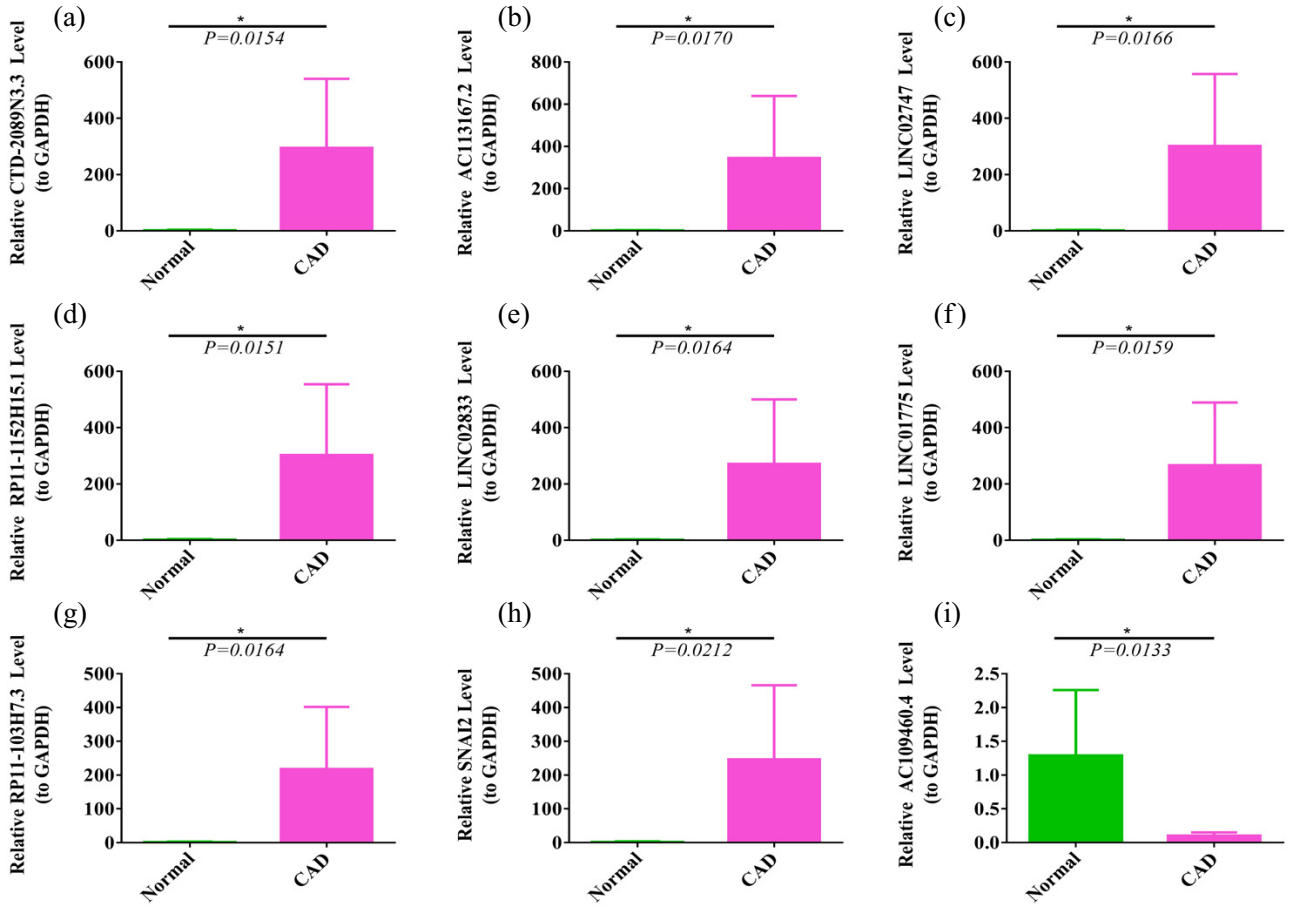
The relative expression of eight EMT-related lncRNAs and SNAI2 in the validation cohort: (a) CTD-2089N3.3, (b) AC113167.2, (c) LINC02747, (d) RP11-1152H15.1, (e) LINC02833, (f) LINC01775, (g) RP11-103H7.3, (h) SNAI2, and (i) AC109460.4.

To assess the accuracy of our results, qPCR was used to detect the expression of eight EMT-related lncRNAs and SNAI2 in peripheral blood samples from six CAD patients and six normal patients (Figure 9). The results showed that the expression patterns of eight EMT-related lncRNAs and SNAI2 were consistent with microarray data in peripheral blood of CAD patients and normal controls. The results show that CTD-2089N3.3 (*P* = 0.0154 < 0.05), AC113167.2 (*P* = 0.0170 < 0.05), LINC02747 (*P* = 0.0166 < 0.05), RP11-1152H15.1 (*P* = 0.0151 < 0.05), LINC02833 (*P* = 0.0164 < 0.05), LINC01775 (*P* = 0.0159 < 0.05), RP11-103H7.3 (*P* = 0.0164 < 0.05), and SNAI2 (*P* = 0.0212 < 0.05) were significantly higher in the CAD groups than in the normal control groups. The expression level of AC109460.4 was increased in patients with normal control groups compared with CAD patients (*P* = 0.0133 < 0.05).

## 4 Discussion

EMT plays a critical physiological and pathological role in developing the cardiovascular system, vascular tissue remodeling, and heart valve disease during the embryonic period [30]. However, more research has been focused on the impact of the EMT in tumor development and treatment. In contrast, few studies have explored the diagnostic value of EMT-related genes or lncRNAs in CAD. Hence, exploring diagnostic biomarkers of EMT-related genes or lncRNAs in CAD is urgent.

Our analyses uncovered 32 EMT-related DEGs in CAD. KEGG pathway analysis of these DE-EMTs was mainly enriched in the PI3K-Akt signaling pathway. Several reports have shown that PI3K-Akt pathway is a canonical EMT signaling pathway [31,32]. Meanwhile, we found this signaling pathway plays an essential role in CAD. A recent study indicated that miRNA-26a-5p activated the PI3K-Akt pathway by targeting phosphatase and tensin homolog (PTEN) and affected the proliferation and apoptosis of endothelial cells isolated from CAD mice [33]. A comparative study also reported that miR-26a-5p could activate the PI3K-Akt signaling pathway through the inhibition of PTEN, thereby protecting against myocardial defect/reperfusion injury [34]. These studies have confirmed that activating the PI3K-Akt signaling pathway can prevent myocardial ischemia-reperfusion in animal models. Other studies have also suggested that regulation of the PI3K-Akt signaling pathway plays a vital role in inhibiting myocardial fibrosis, apoptosis, and the inflammatory response [35,36].

In this study, we performed a coexpression analysis between EMT genes and DElncRNAs through paired lncRNA and mRNA expression data in CAD patients from GEO. Eight differently expressed EMT-related lncRNAs were found to be diagnosis factor for CAD patients. After a literature review, we found no research had been conducted about the mechanisms of the eight lncRNAs except LINC02747. Previous studies have reported that LINC02747 can upregulate the expression of TFE3 by absorbing miR-608 and ultimately promote the proliferation of clear cell renal cell carcinoma (ccRCC) [37]. Gu et al. indicated that miR-608 exerts anti-inflammatory effects by targeting ELANE in monocytes [38]. Our results showed that monocytes were more expressed in the CAD group, so whether the regulation of LINC02747-mir608-ELANE might achieve the reversal of inflammatory response in CAD patients is not clear. Other seven EMT-related lncRNAs have not been reported in relevant studies, and reports on how lncRNAs interact with EMT genes have been rarer. However, many “cis” and “trans” genes are involved in the formation and development of CAD in the cis-trans regulatory network. For example, EMB, as a “cis” gene, was enriched in the mTOR signaling pathway in our GSEA analysis. This pathway is closely associated with atherosclerosis, and the pro-inflammatory response of monocytes in CAD requires activation of mTOR [39]. Among “trans” genes, some studies have reported that VDR gene polymorphisms leads to the development and formation of CAD by affecting changes in serum levels of 25(OH) vitamin D. [40,41]. A previous study reported VDR in regulating inflammation through inhibiting the NF-ĸB pathway and activating autophagy [42]. EBF4 gene promotes the elevation of Cu and leads to the progression of CAD by affecting copper-related DNA methylation sites [43]. CTCF gene is essential for cardiogenesis and to inhibit cardiomyocytes apoptosis, and can be applied as a therapeutic target for the treatment of heart failure in future [44,45]. FLI1 gene is also reported to be closely related to immune dysfunction and platelet disorders [46]. Despite the lack of direct support from literature, we speculated that these cis–trans genes, under the regulation of lncRNA, affect the formation and development of CAD through immune microenvironment, cell apoptosis, platelet dysfunction, and other ways. So far, there has been no study on the role of EMT-related lncRNA in CAD diagnosis. In our study, EMT-related genes with high specificity were identified by bioinformatics methods, and these genes in CAD groups and normal groups were validated by qPCR method. These findings may provide valuable insights into the future diagnosis and treatment of CAD.

The presence of immune cells in the infarct area is vital for initiating the repair process of the injured heart tissue. Temporal and spatial regulation of inflammation after infarction is crucial [47,48]. We evaluated the type and fraction of immune cell infiltration between the CAD patients and normal samples in the data set using the CIBERSORT algorithm. Our results found CD8 T cells and NK cells share a decreased infiltration, and the infiltration of monocytes was increased in CAD patients, which was similar to the previous results [49,50,51]. In this GEO data set, CD8 T cells and NK cells are favorable factors for preventing CAD, and it is likely that monocytes promote the occurrence of CAD. Previous studies have suggested that the imbalance of immune regulation is an essential factor for promoting atherosclerosis, heart failure, and chronic kidney disease by monocytes cells [48]. CD8 T cells play a dual role in atherosclerosis. On the one hand, CD8 T cells can secret many inflammatory cytokines to accelerate the inflammatory response and increase the instability of atherosclerotic plaques. On the other hand, cytotoxic activity against antigen-presenting cells and the presence of regulatory CD8 T cell subsets could suppress immunity and limit atherosclerosis [52]. Ong et al. suggested that NK cells appear to protect the development of cardiac fibrosis by preventing the accumulation of specific inflammatory groups in the heart and directly restricting collagen formation in cardiac fibroblasts [53]. Although the results of our study are similar to these researches, the mechanism of the immune system is still very complex and some results in the immunotherapy of CAD are not ideal. We need a lot of clinical studies to demonstrate the underlying mechanism. Besides, we also found that except AC109460.4, the other seven lncRNAs related to EMT were significantly negatively correlated with CD8 T cell and NK cell and positively correlated with Treg and monocytes. The results of AC109460.4 were just the opposite. The association between these lncRNAs and the innate immune system is still unclear. More *in vivo* and *in vitro* studies are needed to explain the interaction mechanism between these lncRNAs and immune cells in CAD.

It is generally believed that lncRNAs can act in “trans” to regulate TFs mediated chromatin remodeling and transcription [54]. These lncRNAs recruit protein factors to enhancer and regulate enhancer activity [55]. We constructed cis- and trans-regulatory networks based on these eight signatures. In the trans-regulatory network, we obtained 33 differentially expressed TF genes. The most surprising discovery was the screening of SNAI2, an EMT-TF gene (the gene coding product was the transcription factor Snai2). Our results indicated that SNAI2 was not only significantly highly expressed in CAD patients but also strongly positively correlated with LINC01775 and CTD-2089N3.3. The ROC curve showed that the SNAI2 could be a potential biomarker for diagnosing CAD. Additionally, we also validated the high expression of SNAI2 gene in the CAD group by using the qPCR method. As a classic EMT-TF gene, SNAI2 has recently been shown to be involved in a broader range of biological processes, including tumor metastasis, heart development, cell differentiation, vascular remodeling, and DNA damage repair [56,57,58]. Previous studies have reported that the deletion of protein arginine methyltransferase 1 leads to the accumulation of p53, and enhancing the degradation of SNAI2 can limit the formation of cardiac fibroblasts, coronary smooth muscle cells, and pericytes [59]. Meanwhile, Cooley et al. reported that, by grafting mouse veins to the femoral artery in mice to simulate human coronary artery bypass grafting, the results showed that TGF-β/Smad2/3-Snai2-mediated EMT plays a crucial role in venous graft vessel remodeling [60]. These studies have indicated that high expression of SNAI2 can promote the formation of vascular endothelium to EMT and vascular remodeling, which is one of the vital factors in the formation of CAD. At present, the role of SNAI2 in CAD has not been reported. Several studies have proven that the vascular endothelial EMT process is involved in atherosclerosis, post-stent stenosis, pulmonary hypertension, and coronary artery remodeling [61,62,63]. Additionally, the role of EMT can be seen in a range of cell types involved in immunity, such as lymphocytes, NK cells, and myeloid cells, which contribute to inflammatory responses in diverse pathophysiological processes. Ricciardi et al. have reported a decreased viability and proliferation of NK cells and T cells after coculture with cancer cell lines in which EMT had been induced [64]. In our study, SNAI2 correlated with infiltration of monocytes, CD8 T cells, and NK cells was activated. Previous studies have suggested that SNAI2 deletion in mice leads to impaired development of the T-lymphatic system [65]. Subsequent studies also confirmed that Snai2 plays a vital influence in regulating CD8 T cells and targets genes with functions for T cells [66]. Furthermore, our results indicated that the difference in these immune cell infiltrations in the SNAI2 high expression group was similar to the results of CAD patients. These immune cells have been researched to play a role in the formation, erosion, and rupture of coronary plaques [67,68]. In summary, we inferred that SNAI2 might play a significant role in the occurrence of CAD by regulating innate and adaptive immunity through these immune cells. To confirm our conclusion, more experimental mechanistic research should be carried out in future studies.

Our study should acknowledge some limitations. First, these EMT-related lncRNAs were investigated in data sets with no access to individual patients’ characteristics; thus, we cannot adjust the ROC curve for traditional cardiovascular risk factors. A prospective cohort recruiting CAD patients is needed to confirm the predictive value of EMT-related lncRNAs. Second, the MF details of SNAI2 and EMT-related lncRNAs in the progression of CAD have not been further studied. Therefore, molecular biological experiments and flow cytometry analysis are required to validate these findings, and another external validation based on a larger sample is needed.

## 5 Conclusion

In conclusion, this comprehensive bioinformatic analysis revealed that SNAI2 and EMT-related lncRNAs could be reliable biomarkers for diagnosing CAD and may be used for decision-making in the treatment of CAD patients. At the same time, based on the eight EMT-related lncRNAs, we constructed the cis- and trans-regulatory networks of CAD. Furthermore, the immune analysis suggested that these biomarkers were closely related to immune cells and CAD. These findings provide references for clinicians to understand the molecular mechanism of interaction between CAD and EMT and develop individualized treatment for CAD patients.

## Abbreviations

CAD: coronary artery disease
EMT: epithelial-mesenchymal transition
lncRNAs: long-coding RNAs
MALAT1: metastasis associated lung adenocarcinoma transcript 1
miRNAs: microRNAs
SNPs: single nucleotide polymorphisms
SVM-RFE: support vector machine reverse feature elimination
GSEA: gene set enrichment analysis
ssGSEA
BP: biological processes
MF: molecular function
CC: cellular components
KEGG: kyoto encyclopedia of genes and genomes
DEGs: differentially expressed genes
DE-EMTs: differentially expressed EMT genes
DElncRNAs: differentially expressed lncRNAs
FC: fold-change
CIBERSORT: cell type identification by estimating relative subsets of RNA transcripts algorithm
PCA: principal component analysis
ccRCC: clear cell renal cell carcinoma
GEO: gene expression omnibus
LASSO: least absolute shrinkage and selection operator
FDR: false discovery rate
AUC: the area under the curve
ROC: receiver operating characteristic
PRMT1: protein arginine methyltransferase 1
CABG: coronary artery bypass grafting.

## Supplementary Material

Supplementary Figure 1

Supplementary Figure 2

Supplementary Figure 3

Supplementary Figure 4A

Supplementary Figure 4B

Supplementary Figure 5

Supplementary Figure 6A

Supplementary Figure 6B

Supplementary Figure 6C

Supplementary Figure 6D

Supplementary Figure 6E

Supplementary Figure 6F

Supplementary Figure 6G

Supplementary Figure 6H

Supplementary Figure 6I

Supplementary Table 1

Supplementary Table 2

Supplementary Table 3

Supplementary Table 4

Supplementary Table 5

Supplementary Table 6

Supplementary Table 7

Supplementary Table 8
