# Supplementary figures and images for: Epithelial-mesenchymal transition-related genes in coronary artery disease
Source: Open Med (Wars). 2022 Apr 22;17(1):781–800. doi: 10.1515/med-2022-0476 (PMC9034345; doi:10.1515/med-2022-0476)

**A**

CTD-2089N3.3

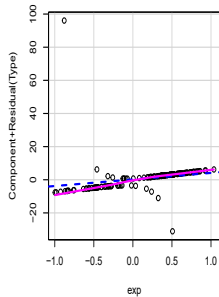

AC113167.2

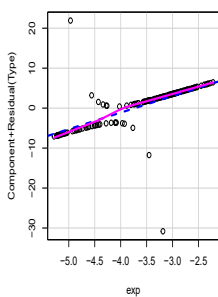

LINC02747

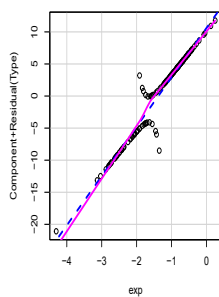

RP11-1152H15.1

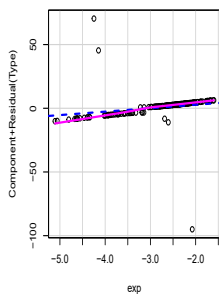

LINC02833

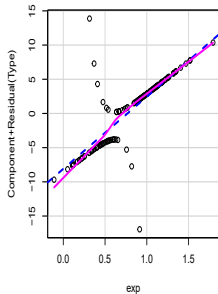

AC109460.4

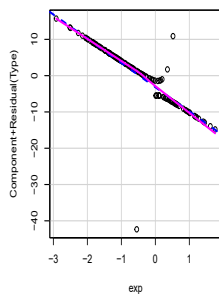

LINC01775

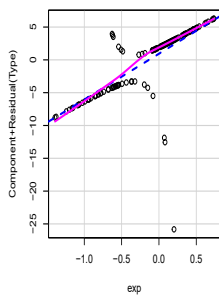

RP11-103H7.3

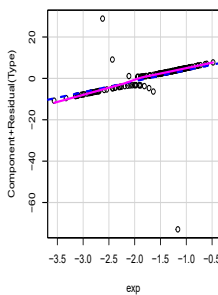**B**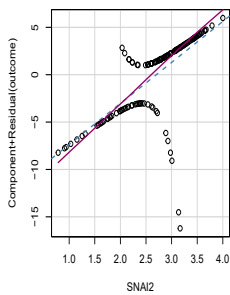

Supplement: Supplementary Figure 1 [file med-2022-0476-Fig-S1.pdf]

## LncLocator

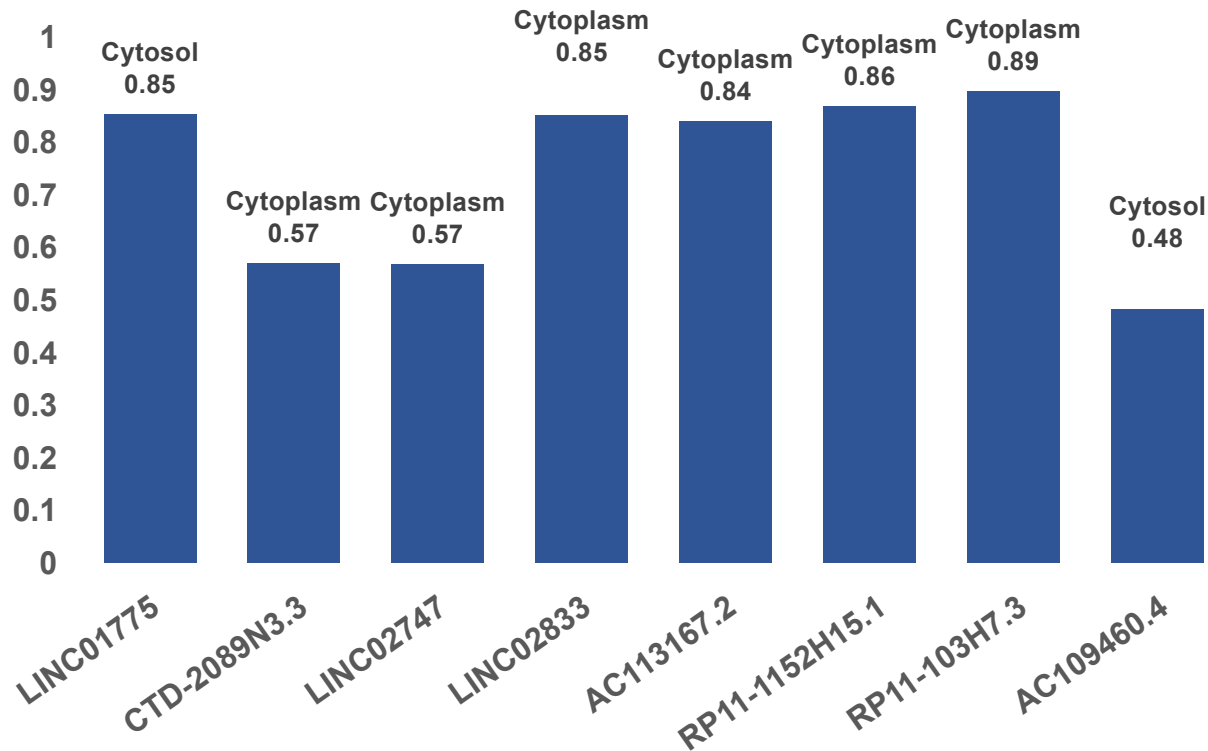

Supplement: Supplementary Figure 2 [file med-2022-0476-Fig-S2.pdf]

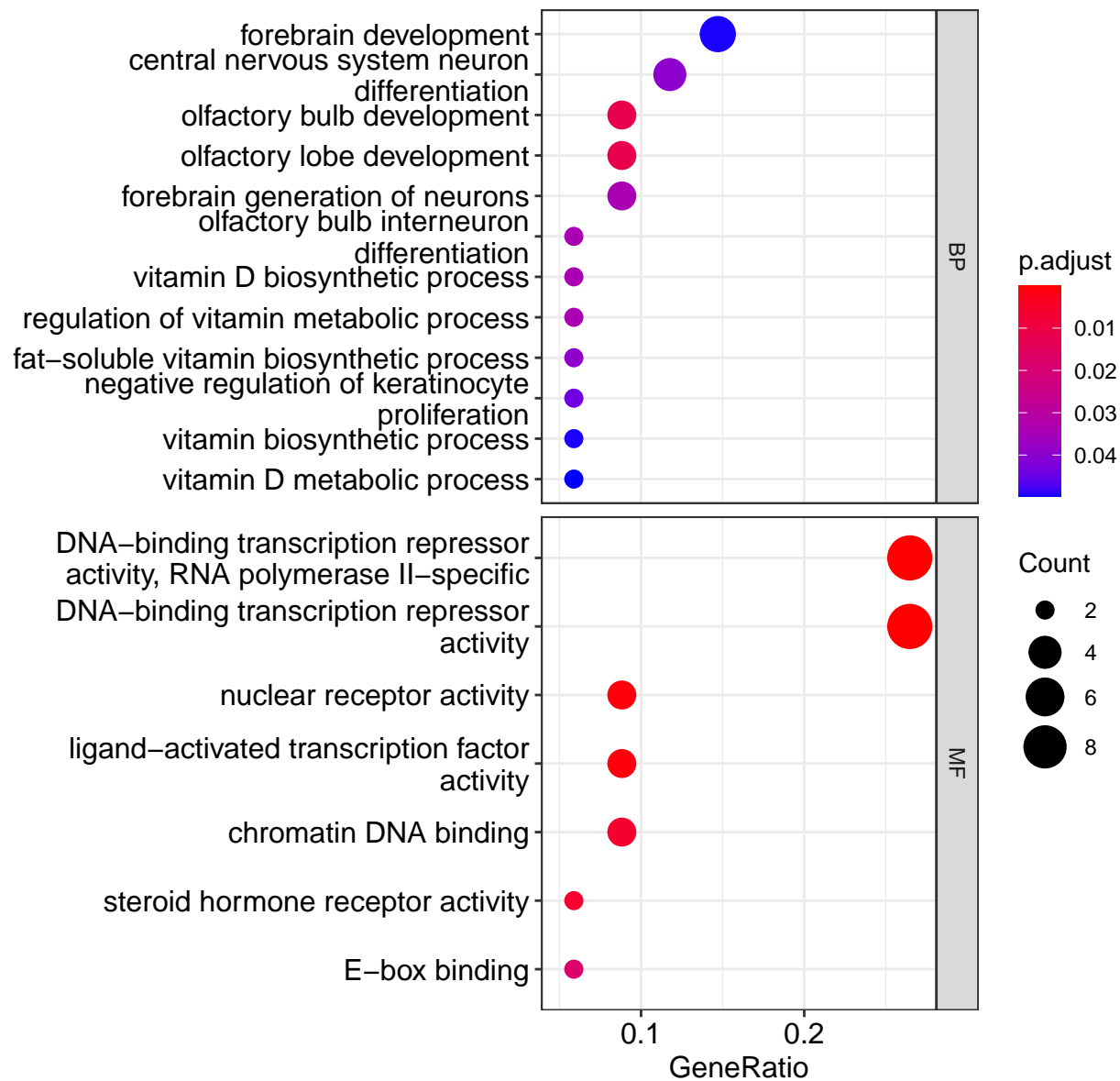

Supplement: Supplementary Figure 4A [file med-2022-0476-Fig-S4A.pdf]

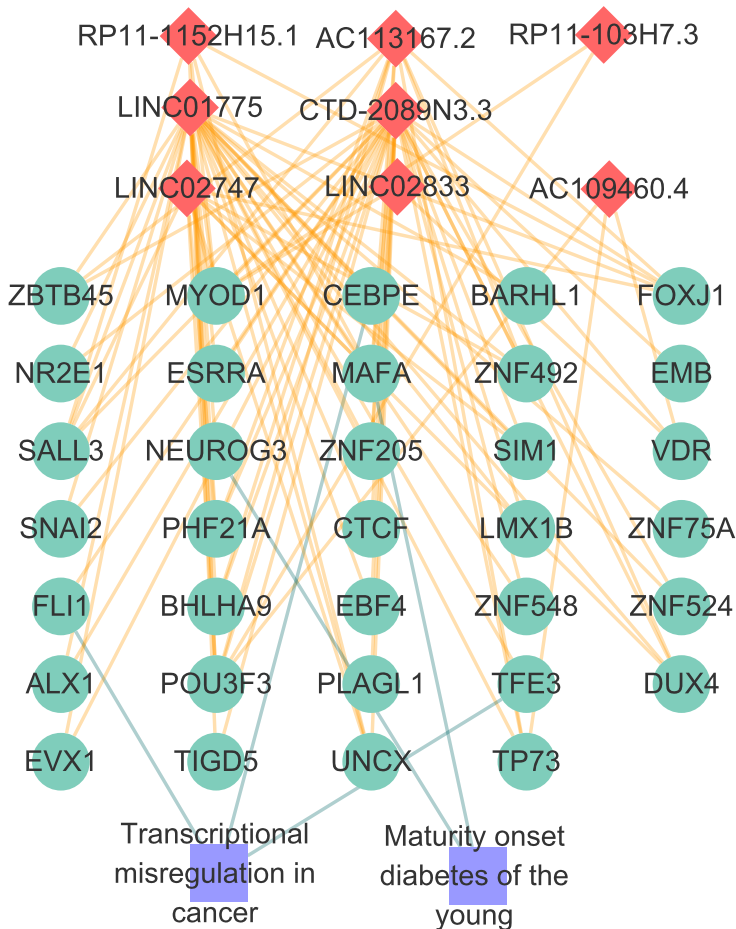

Supplement: Supplementary Figure 4B [file med-2022-0476-Fig-S4B.pdf]

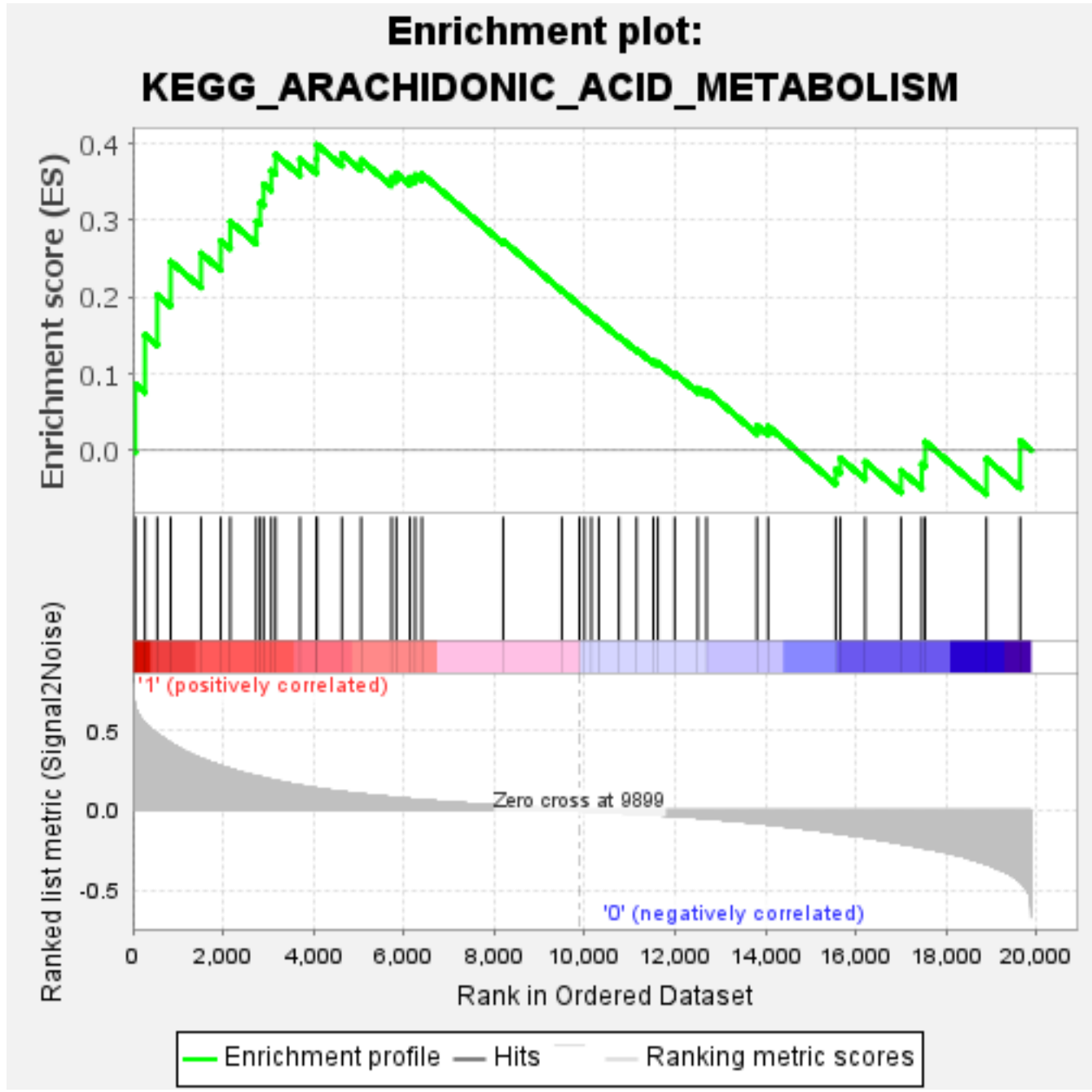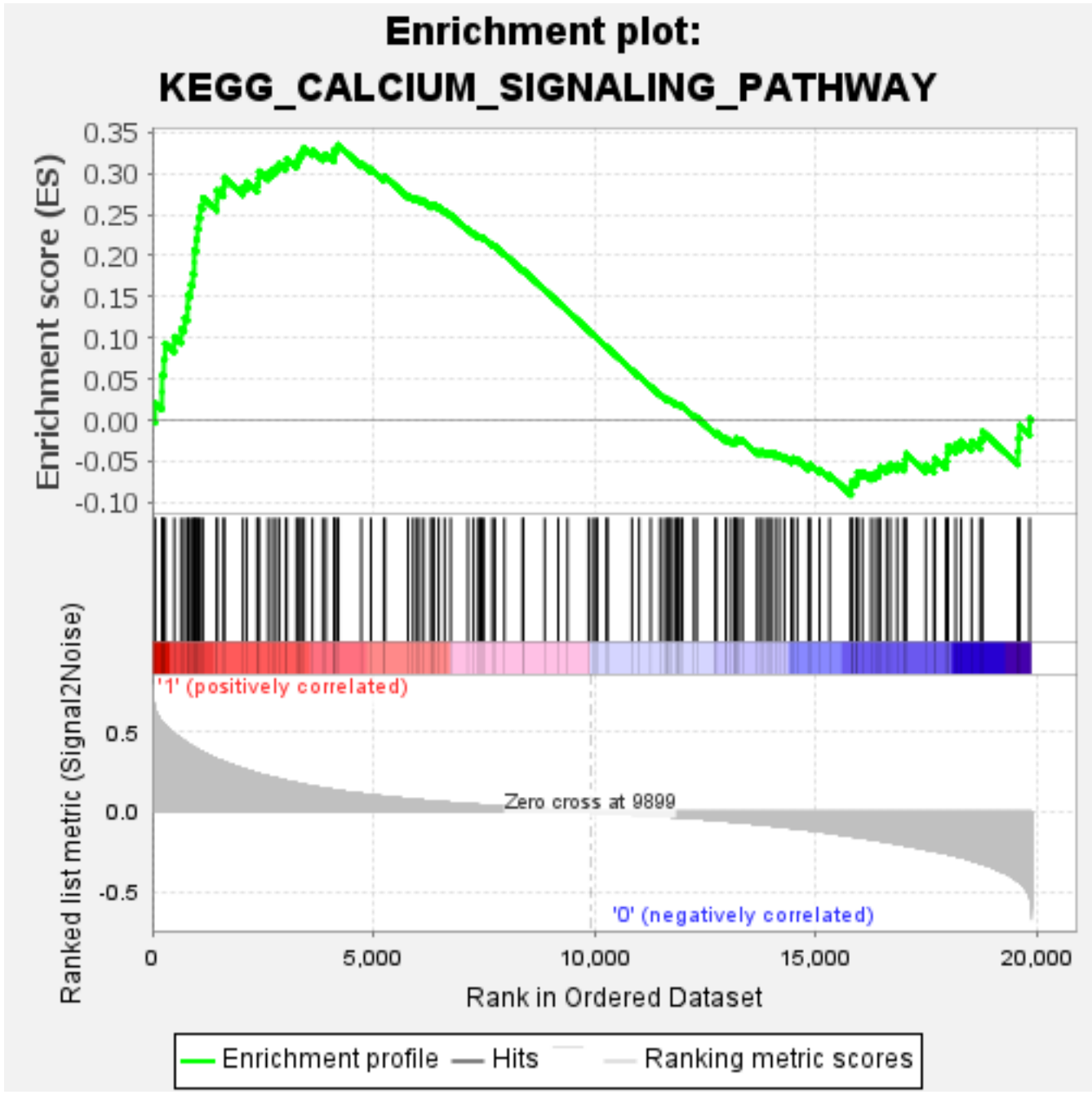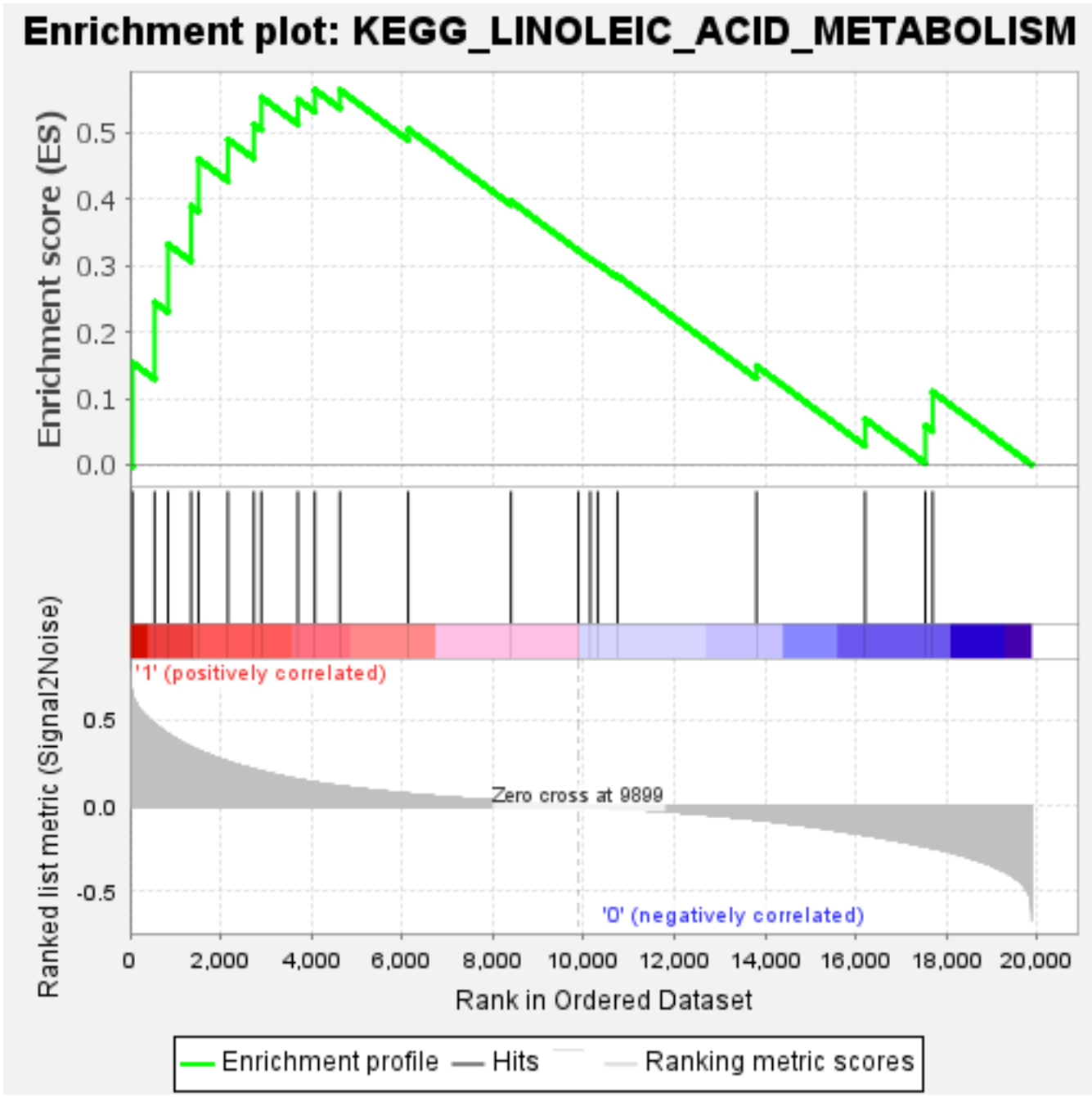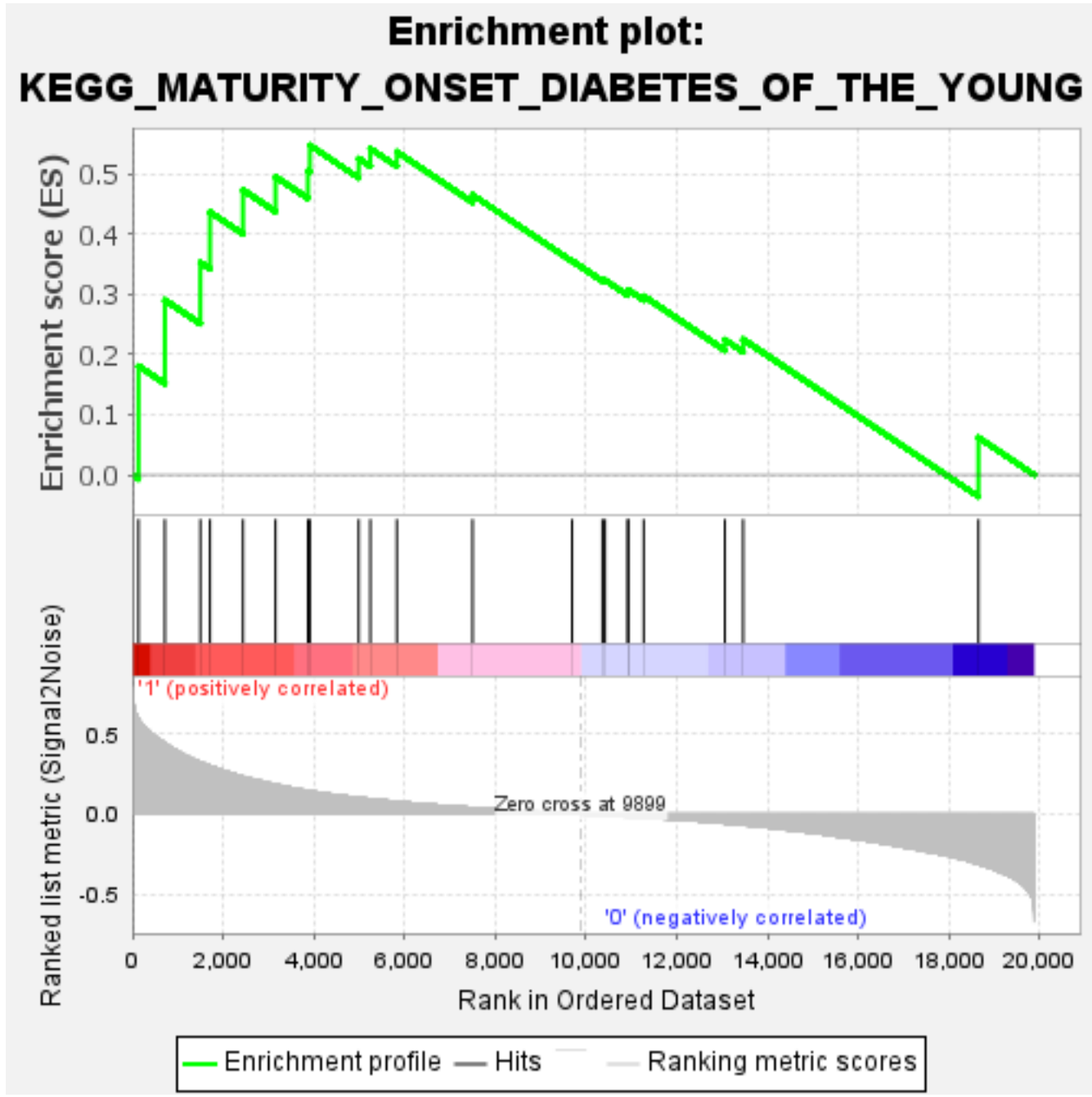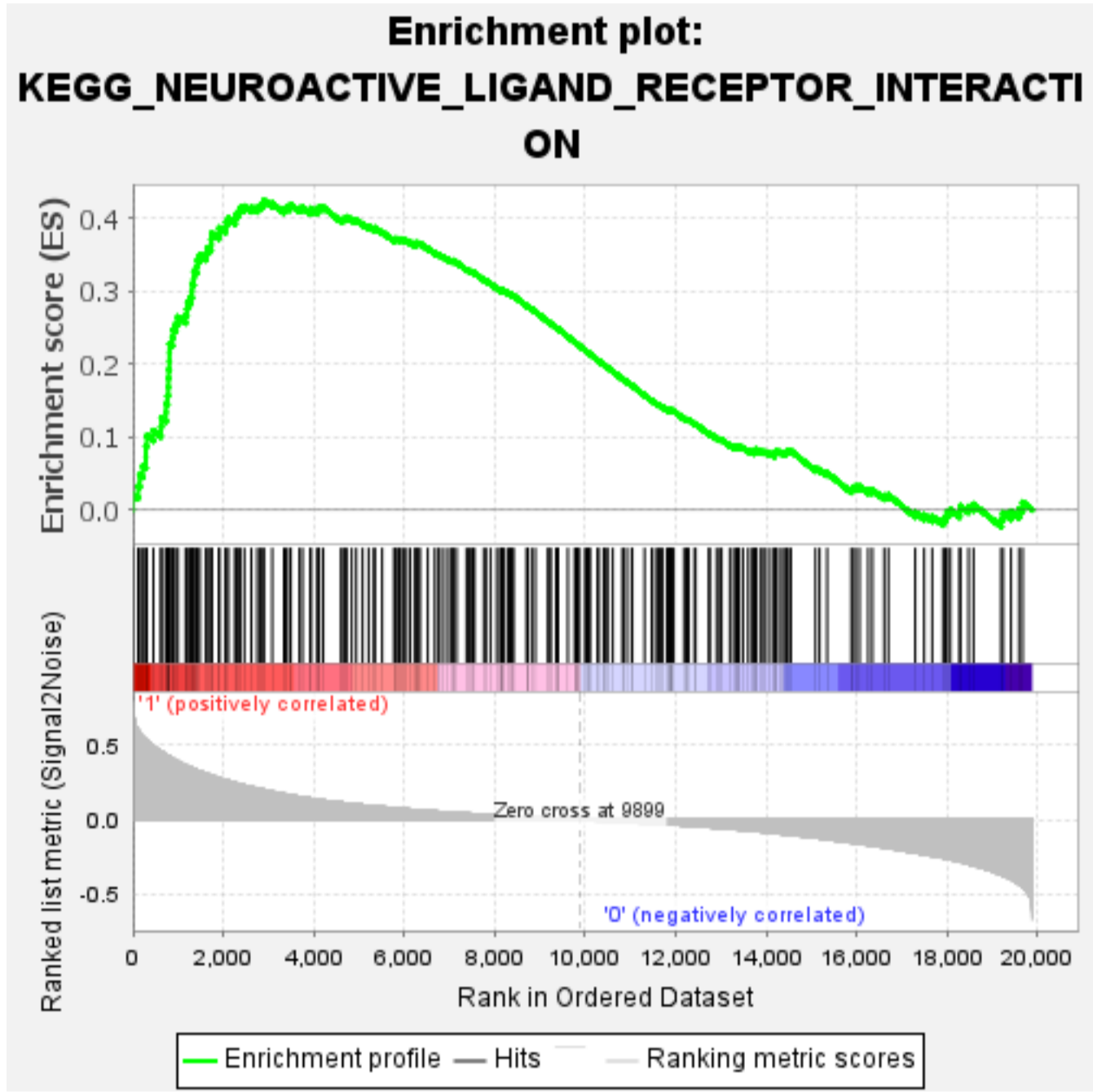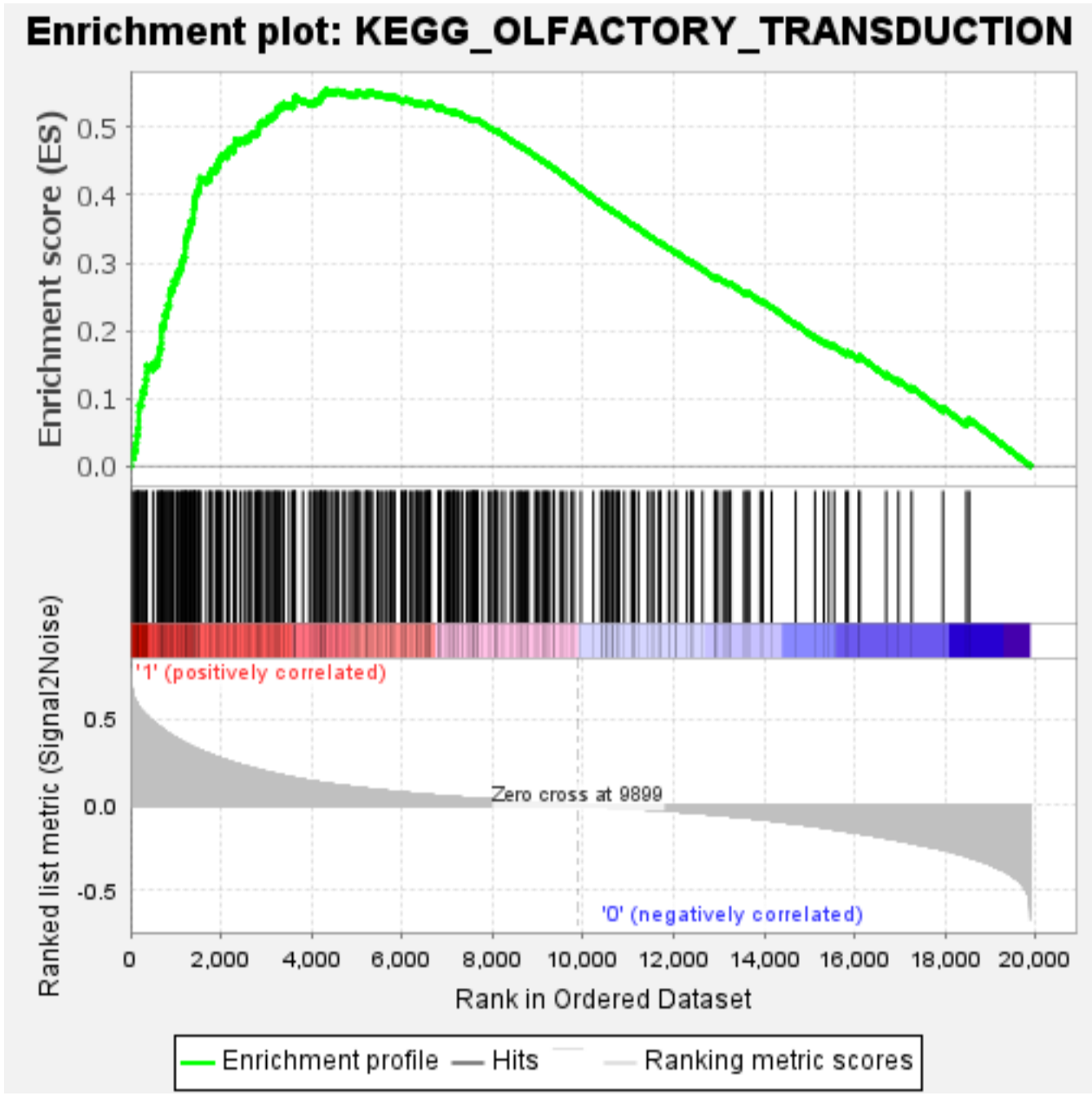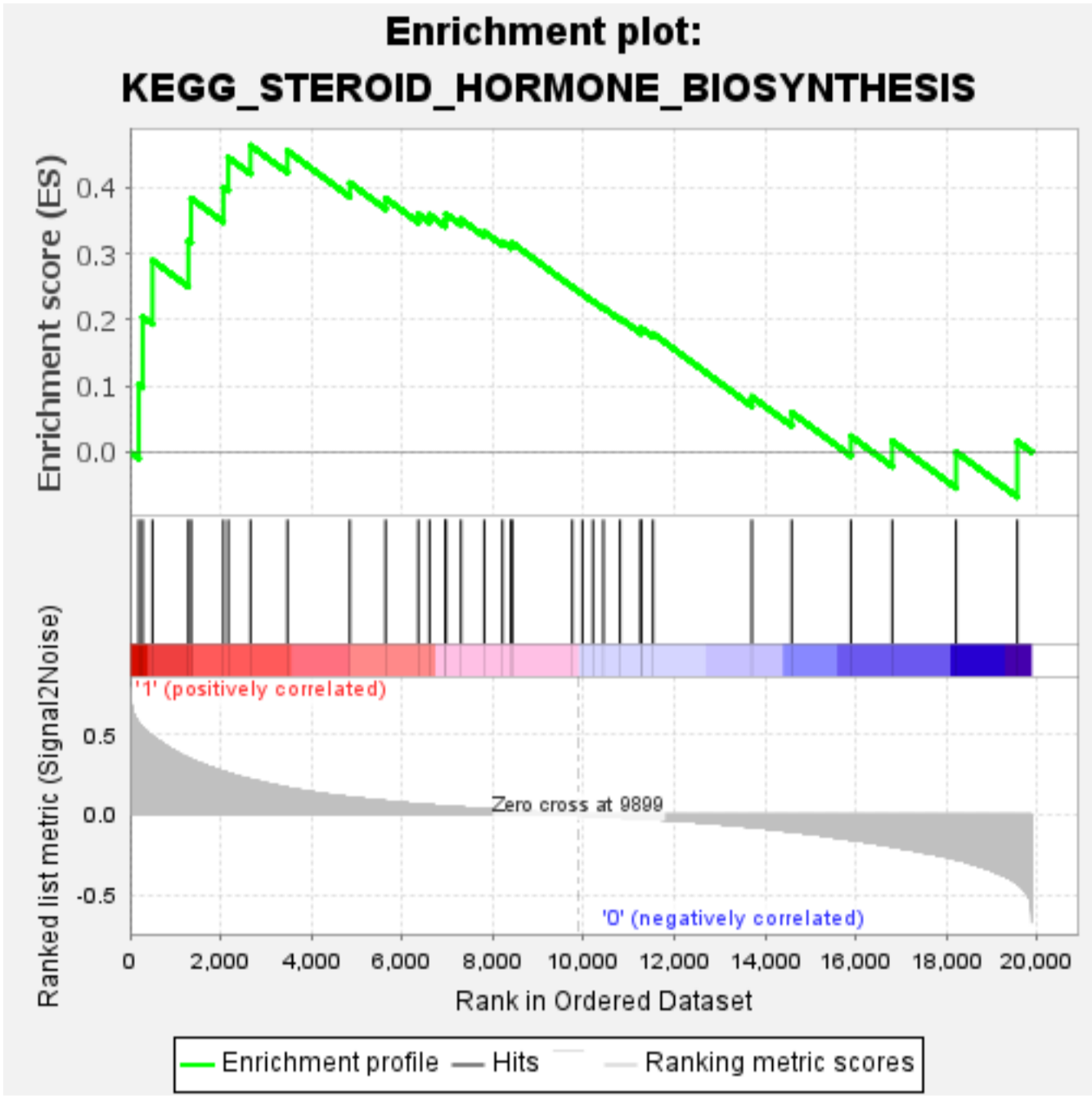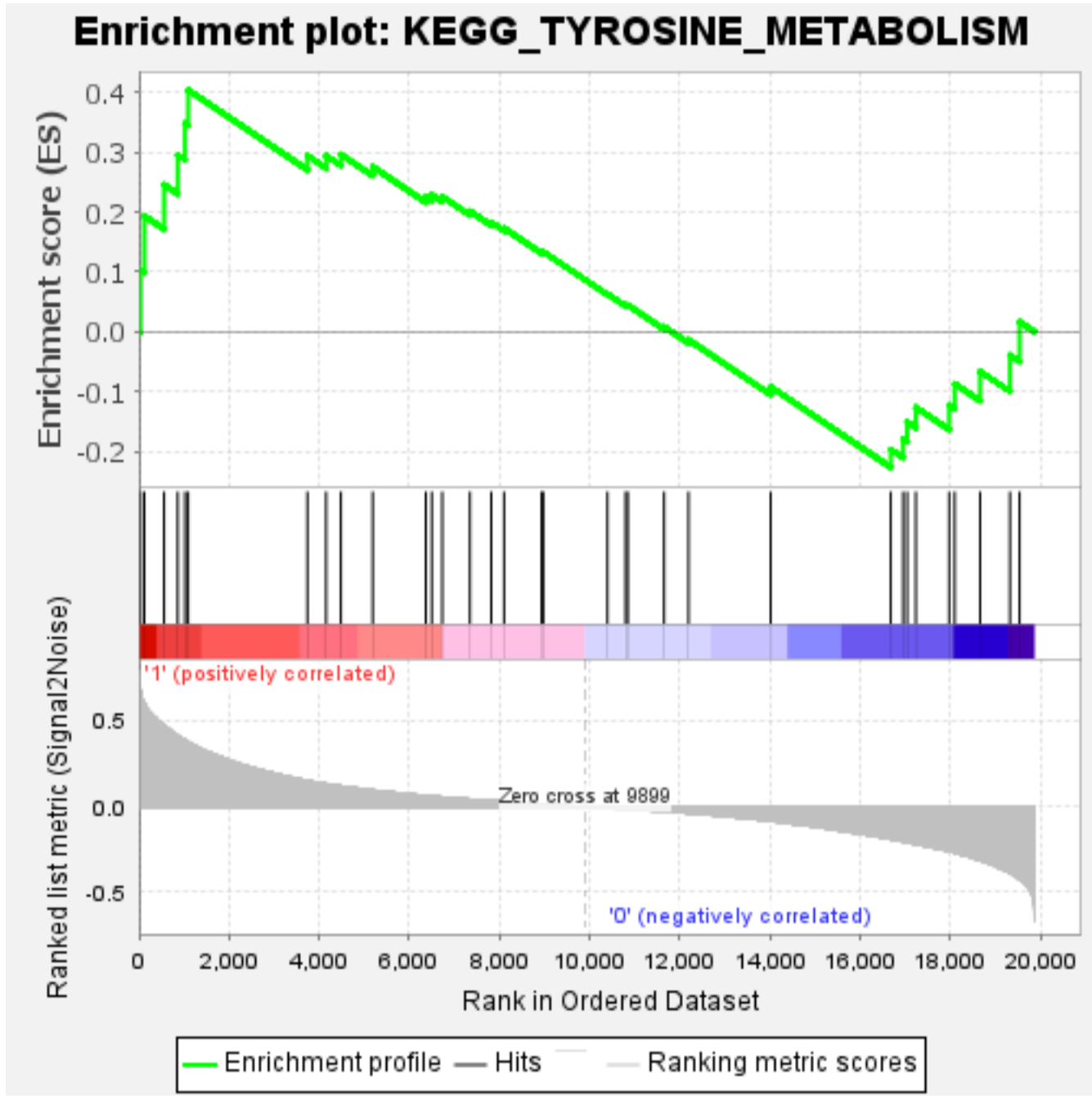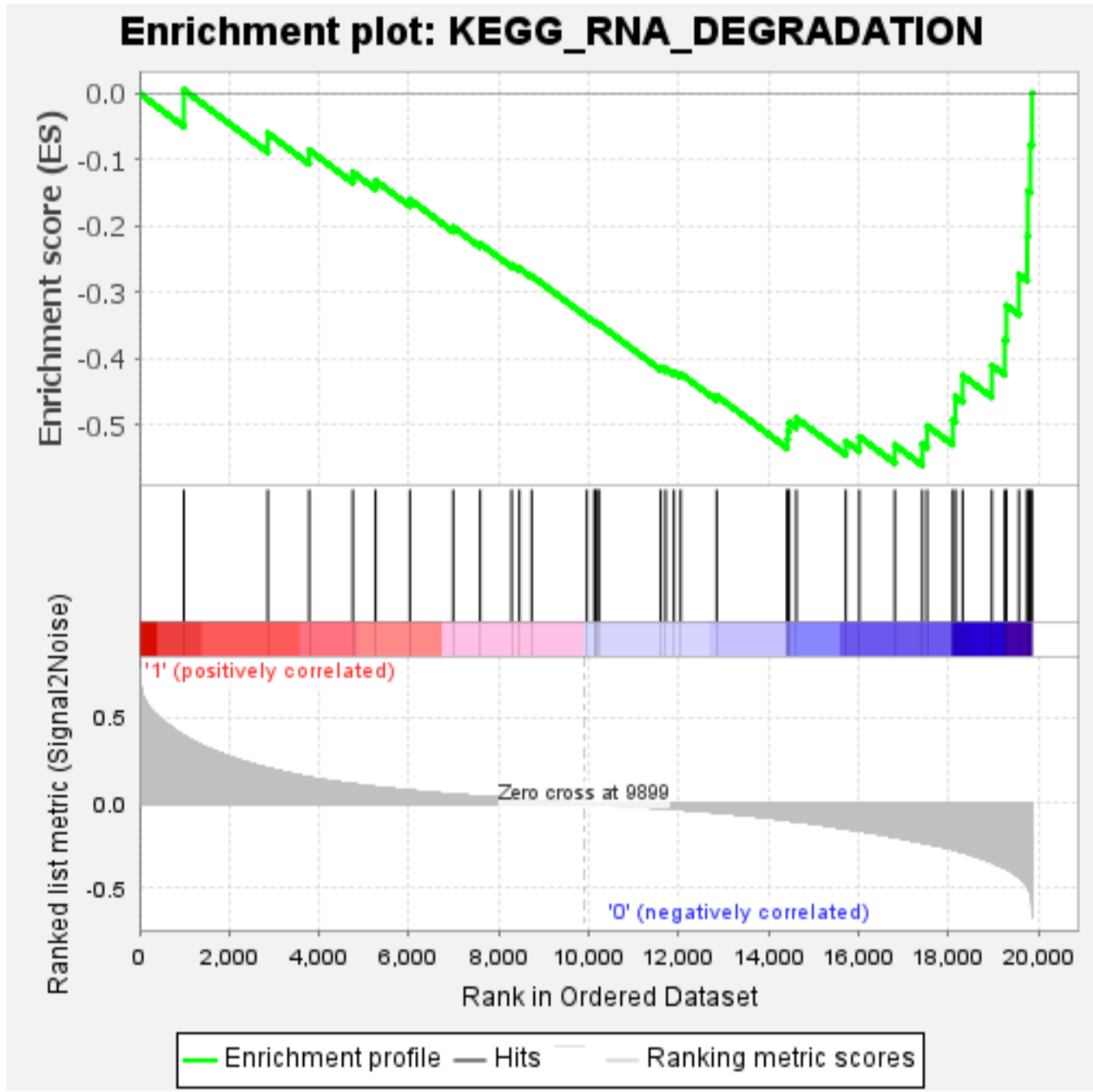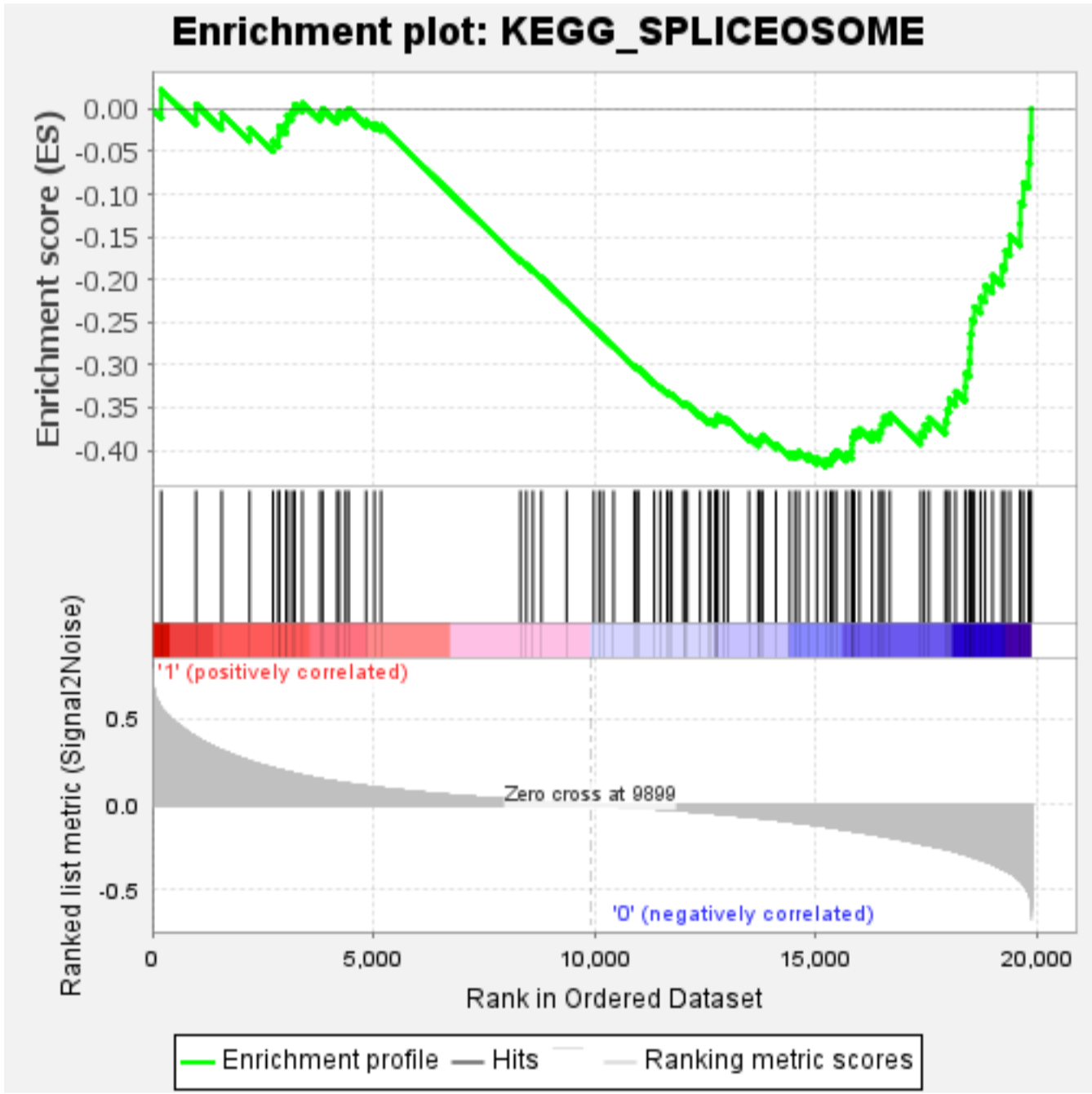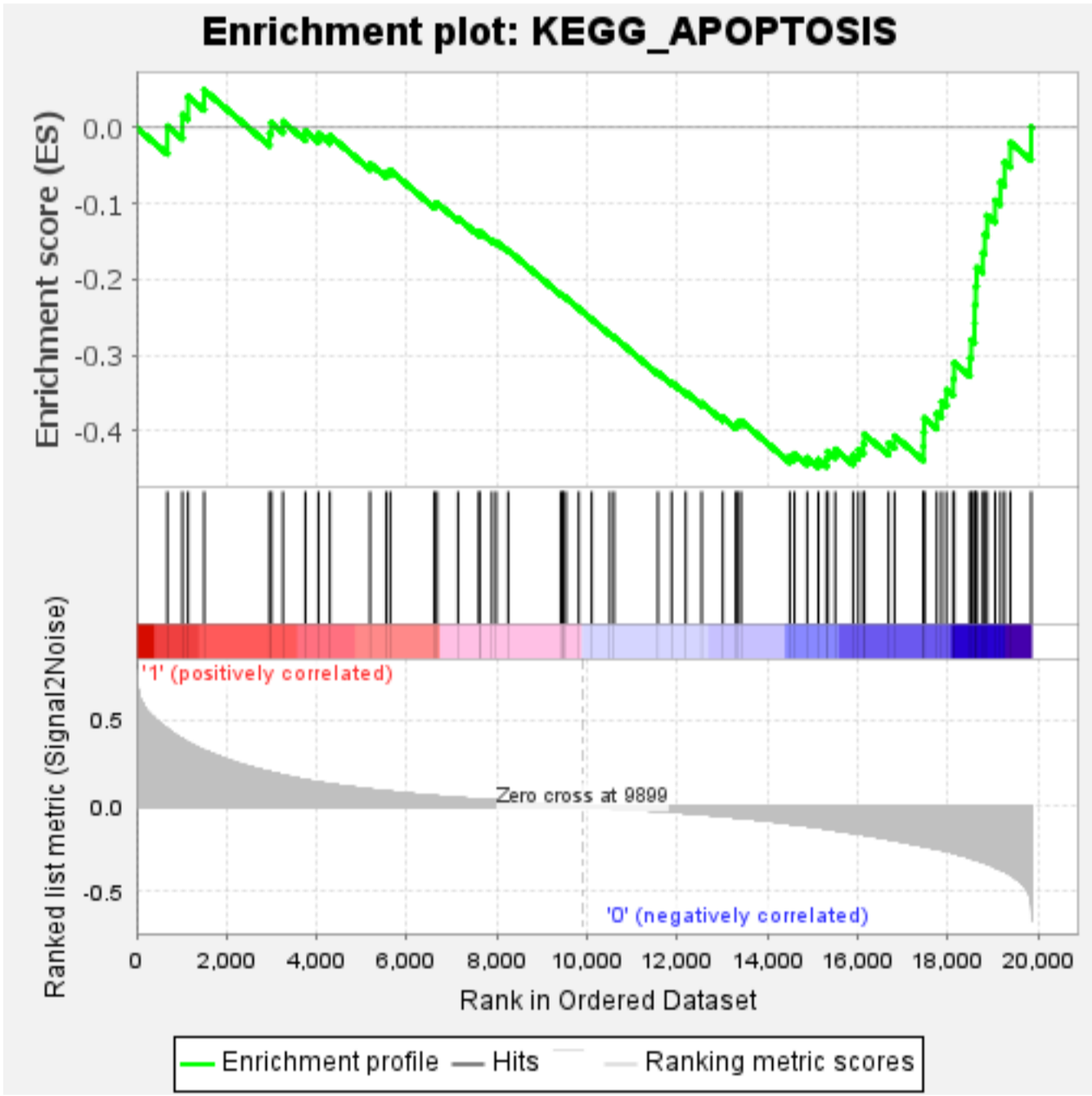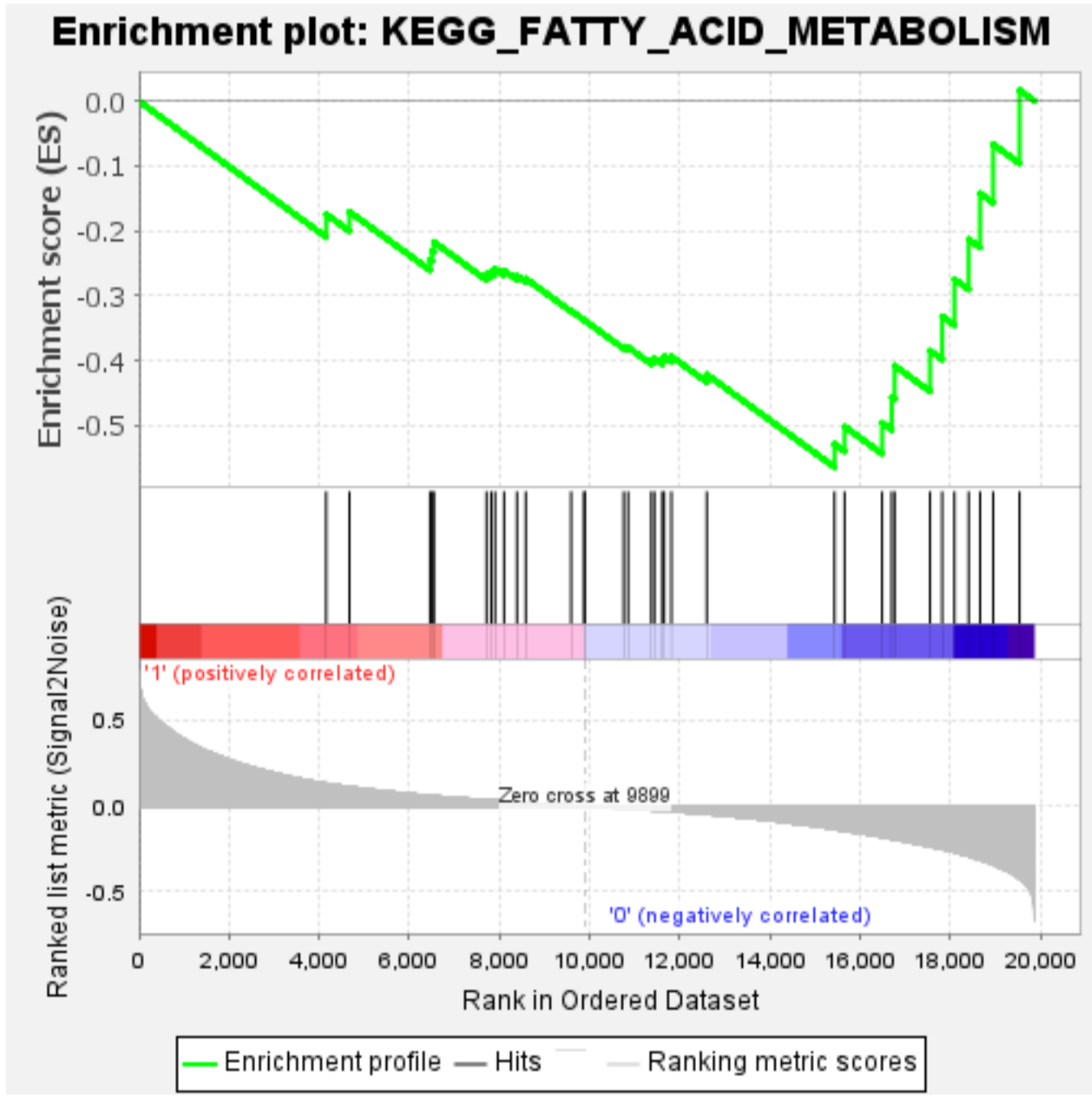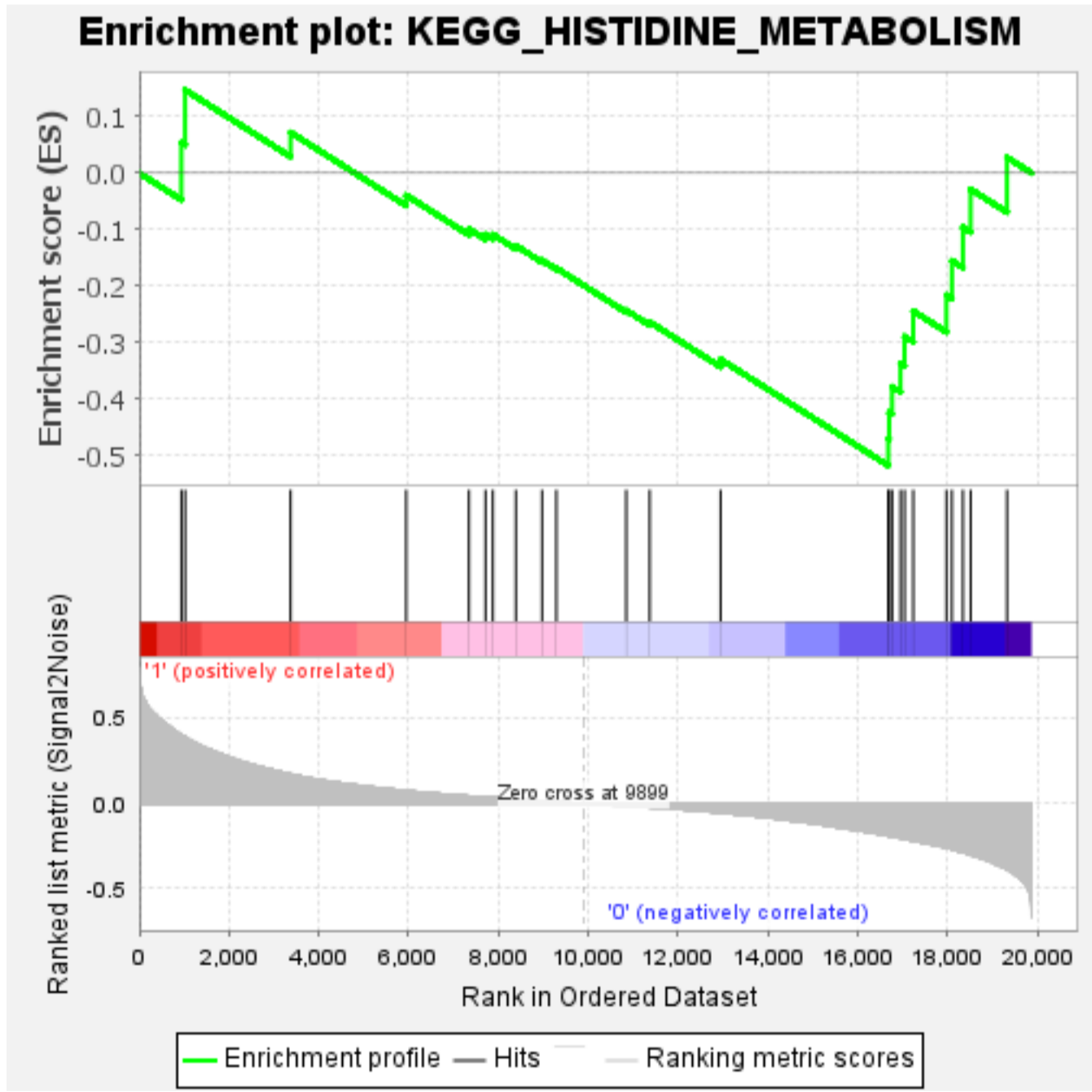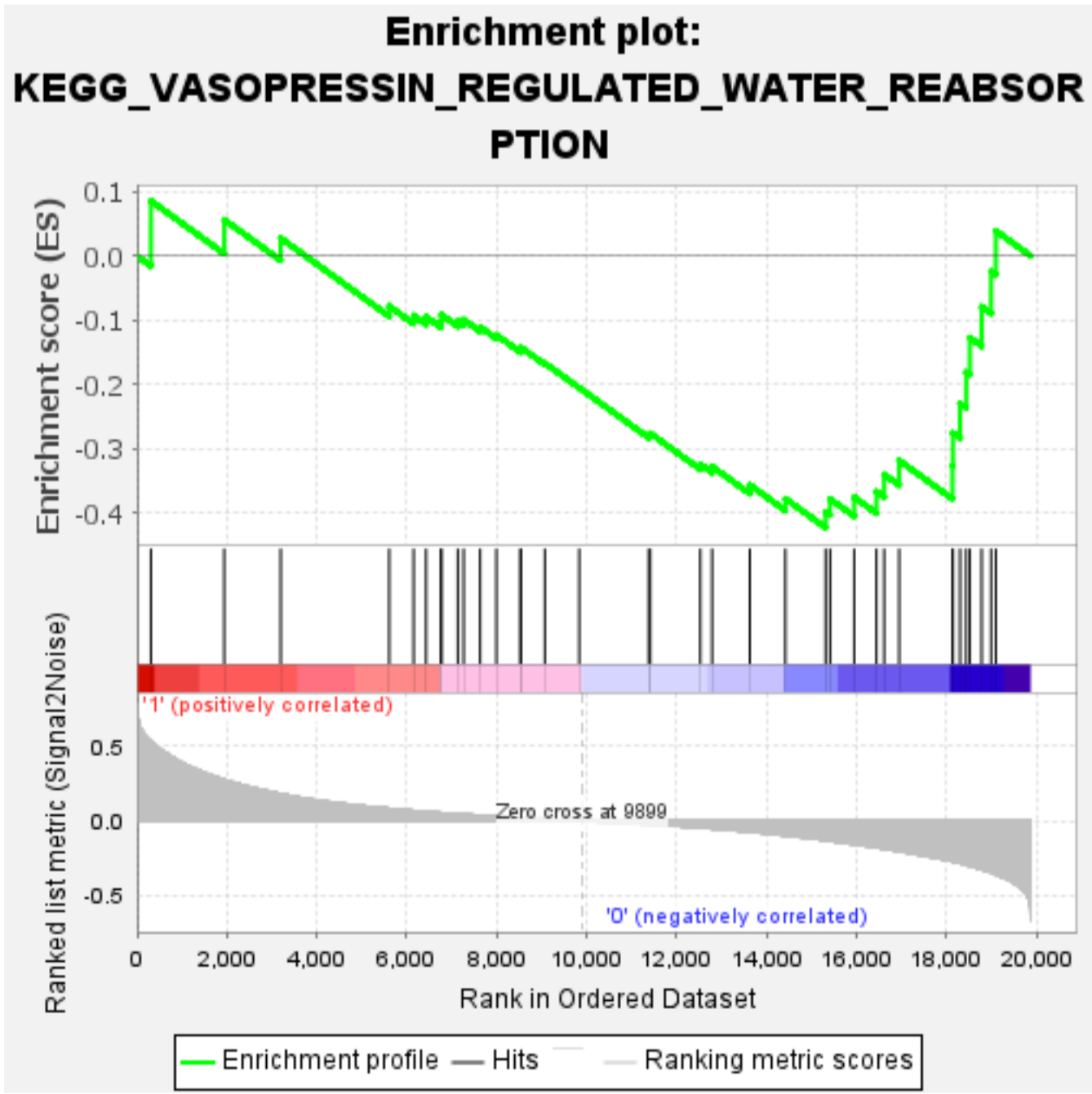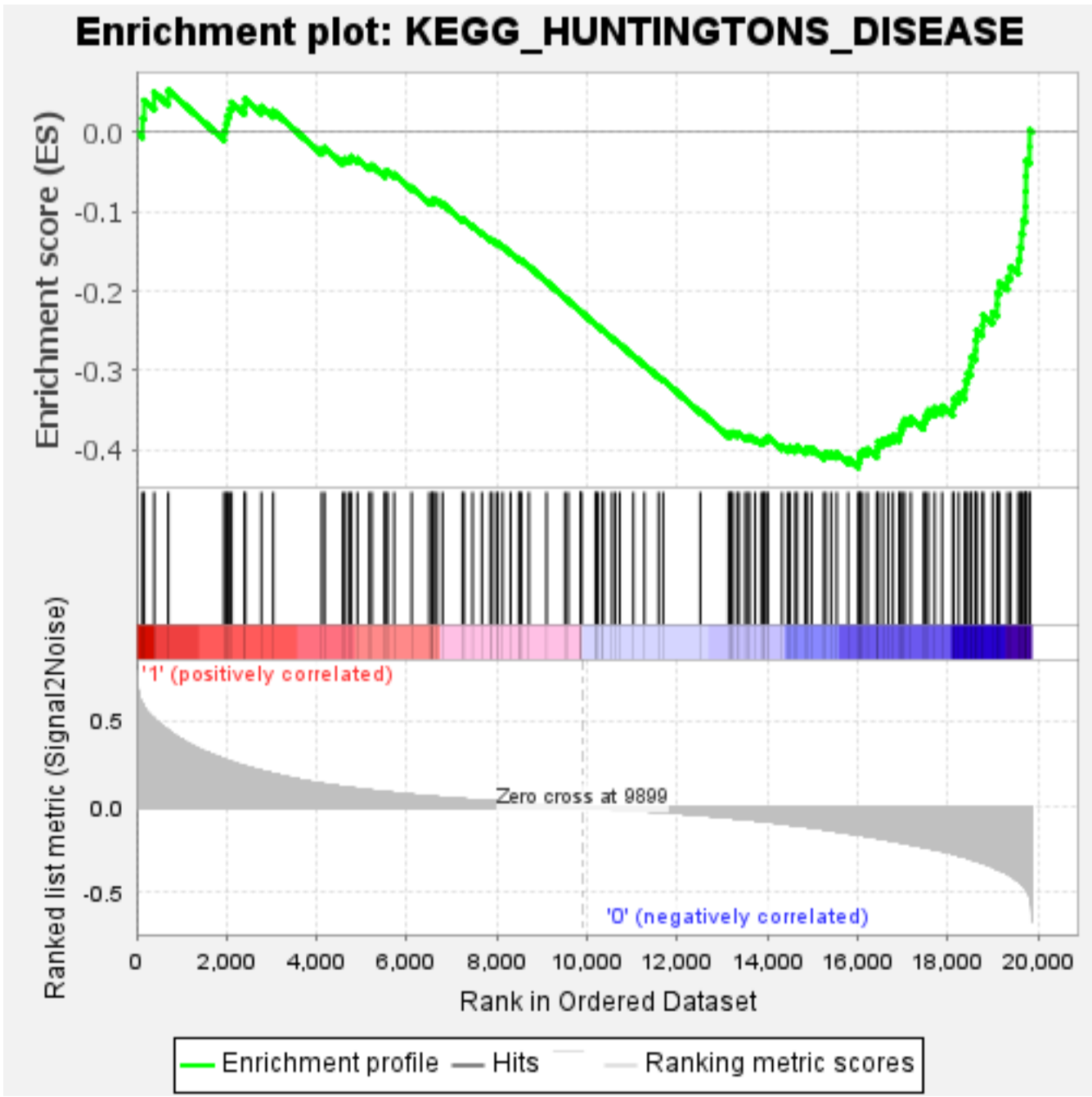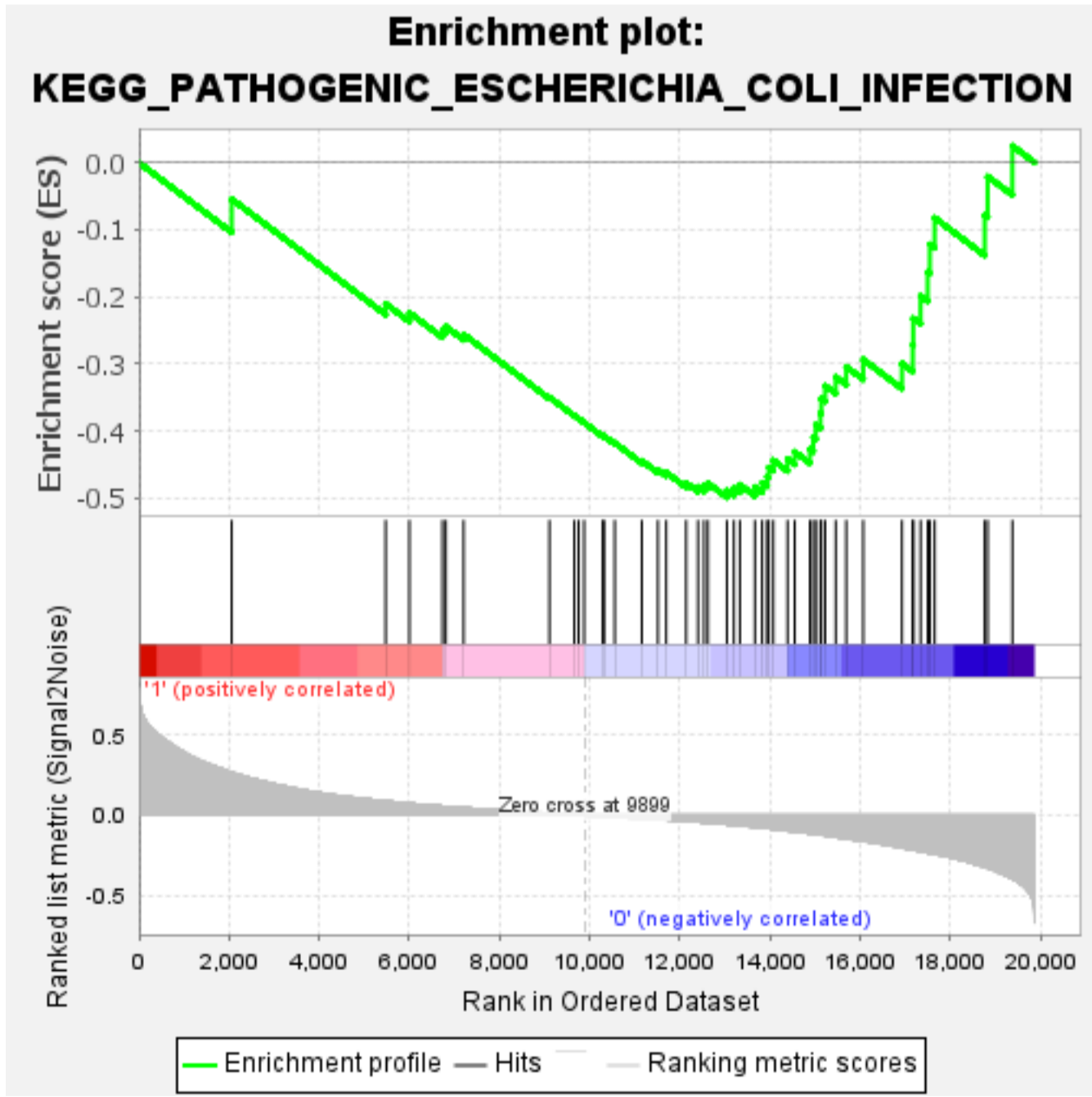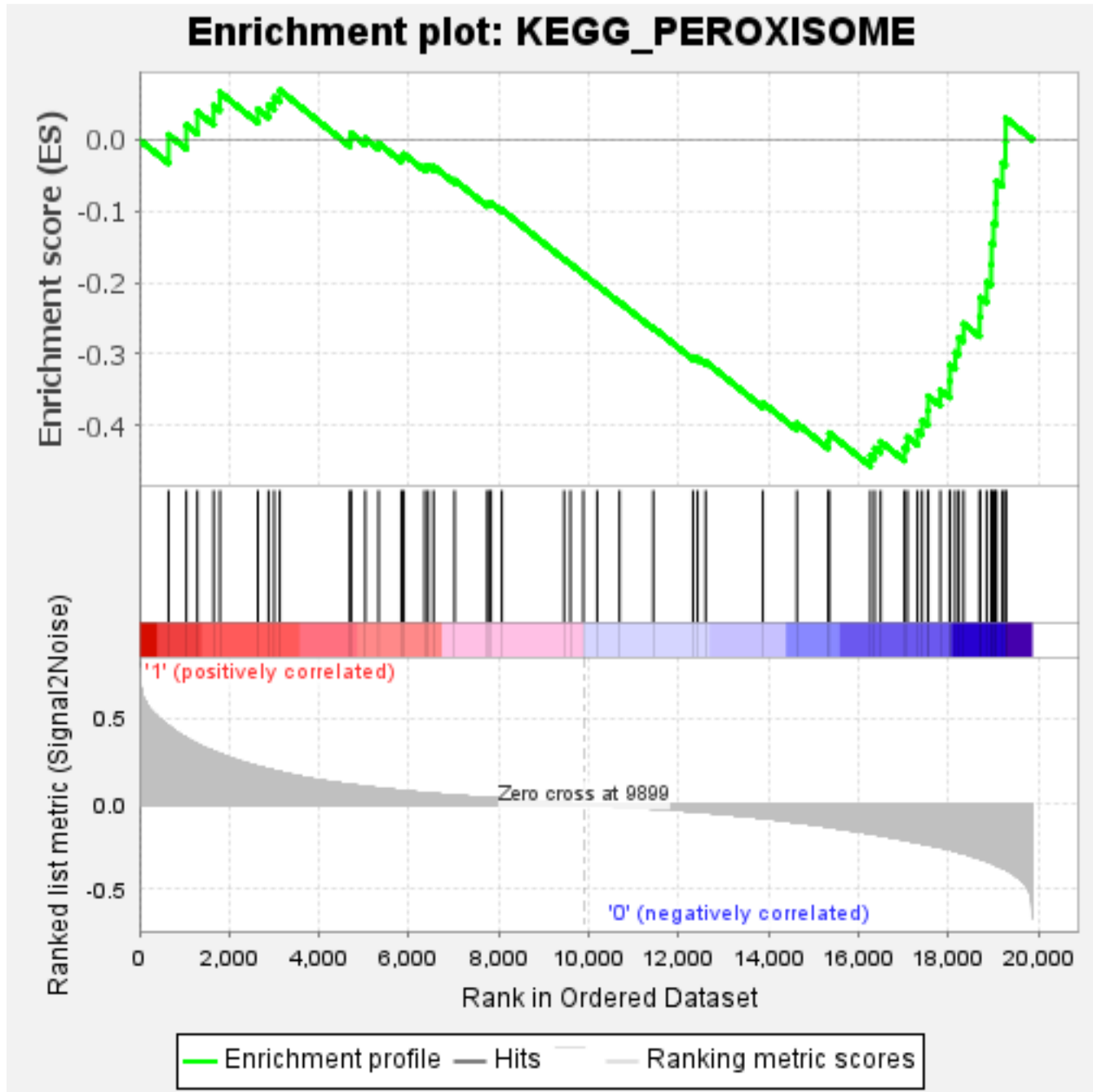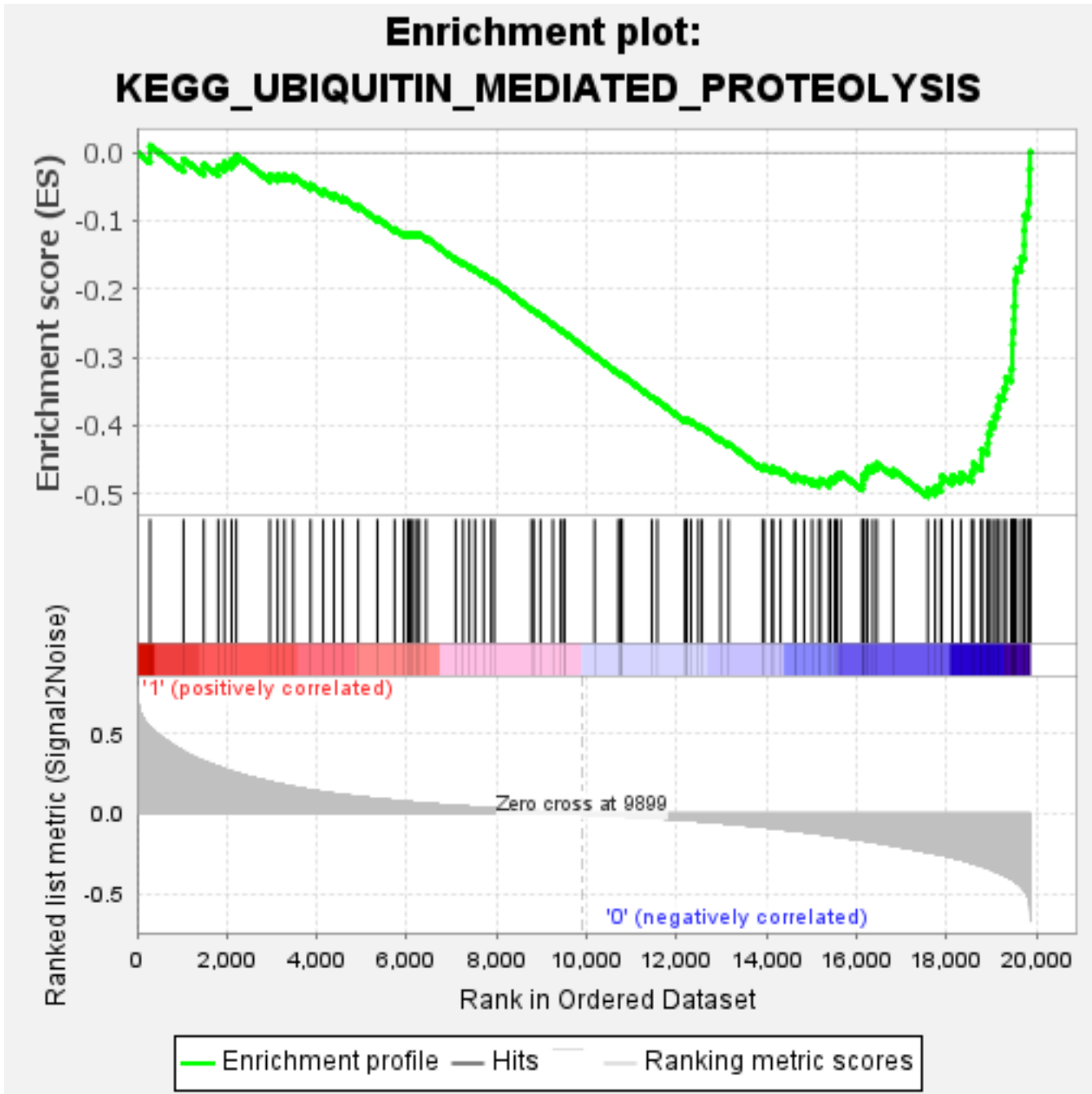

Supplement: Supplementary Figure 5 [file med-2022-0476-Fig-S5.pdf]

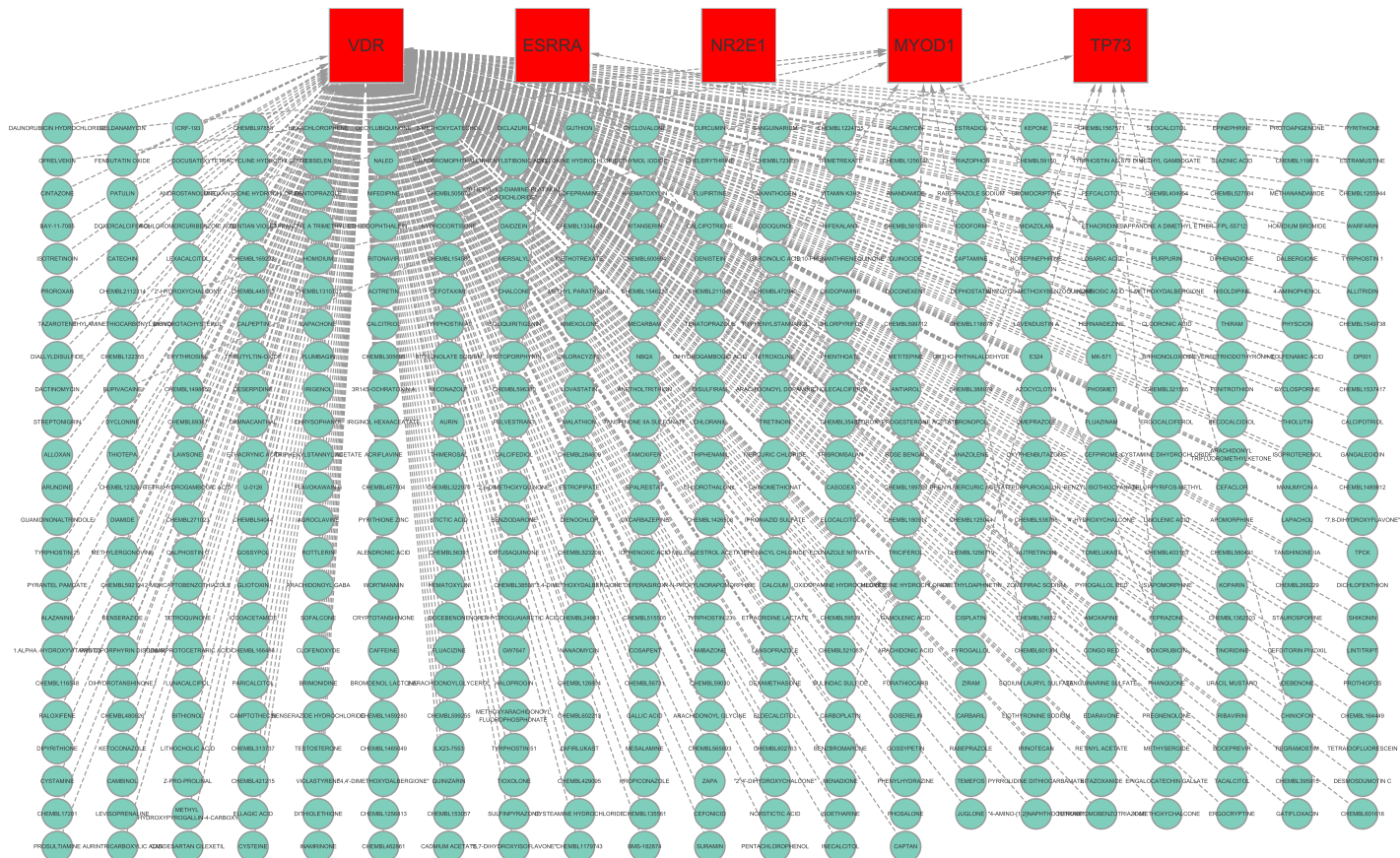

Supplement: Supplementary Figure 6A [file med-2022-0476-Fig-S6A.pdf]
