# Supplementary material for: Epithelial-mesenchymal transition-related genes in coronary artery disease
Source: Open Med (Wars). 2022 Apr 22;17(1):781–800. doi: 10.1515/med-2022-0476 (PMC9034345; doi:10.1515/med-2022-0476)

DAIDZEIN

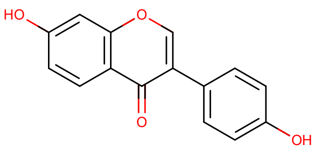

GENISTEIN

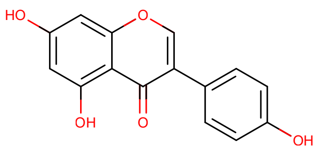

PREGNENOLONE

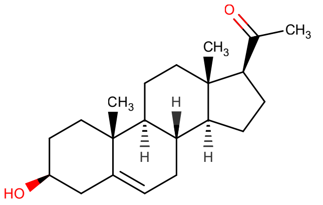

RIMEXOLONE

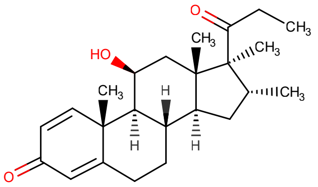

FULVESTRANT

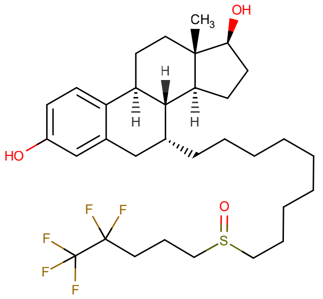

ESTRADIOL

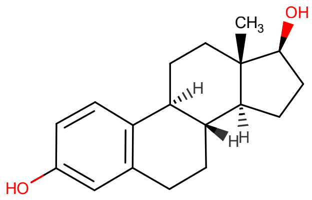

CLODRONIC ACID

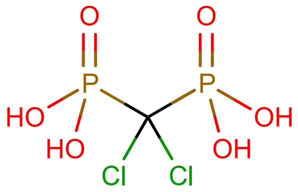

DOXORUBICIN

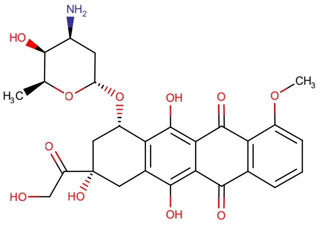

GOSERELIN

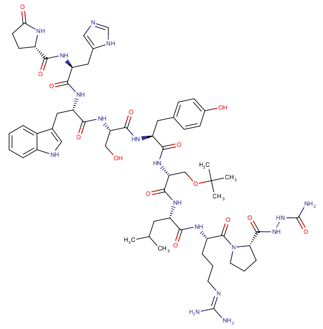

ANDROSTANOLONE

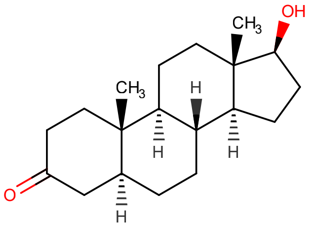

LIOTHYRONINE SODIUM

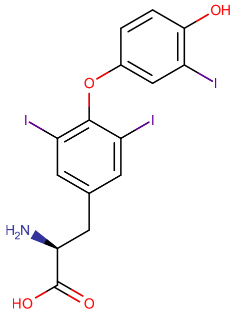

TAMOXIFEN

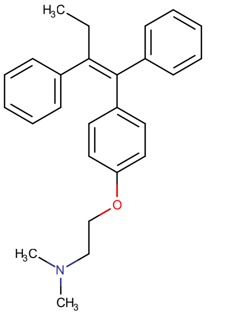

BUPIVACAINE

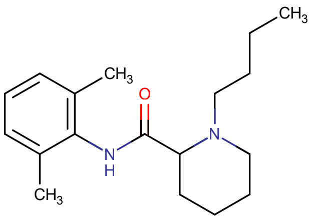

OPRELVEKIN

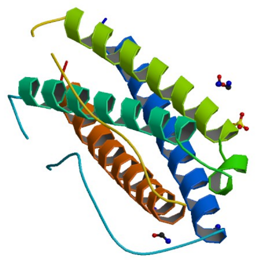

GELDANAMYCIN

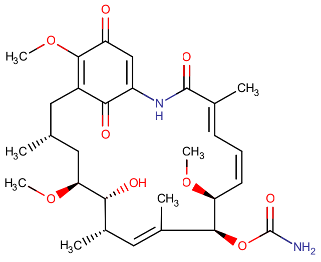

TRETINOIN

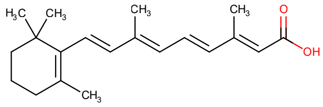

CISPLATIN

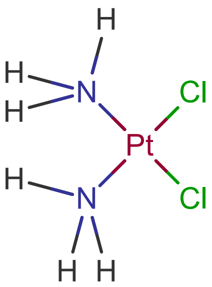

CARBOPLATIN

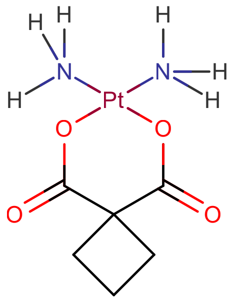

TAZAROTENE

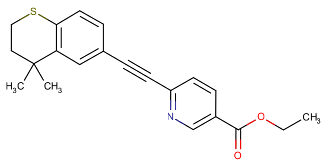

CHOLECALCIFEROL

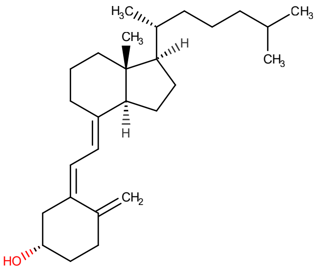

SEOCALCITOL

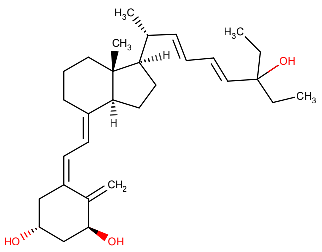

DOXERCALCIFEROL

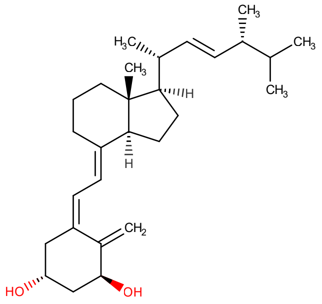

PARICALCITOL

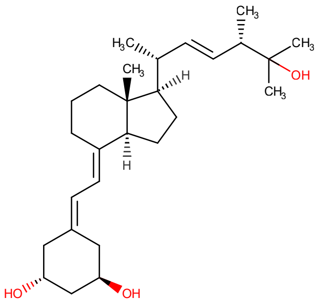

TACALCITOL

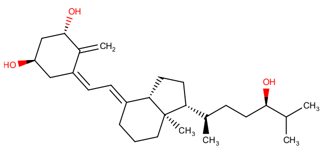

Supplement: Supplementary Figure 6B [file med-2022-0476-Fig-S6B.pdf]
