# Supplementary material for: Epithelial-mesenchymal transition-related genes in coronary artery disease
Source: Open Med (Wars). 2022 Apr 22;17(1):781–800. doi: 10.1515/med-2022-0476 (PMC9034345; doi:10.1515/med-2022-0476)

DIHYDROTACHYSTEROL

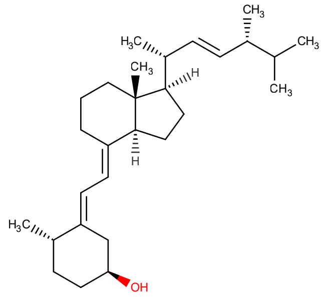

CALCIPOTRIOL

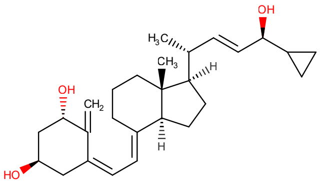

LEXACALCITOL

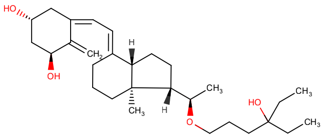

CALCIFEDIOL

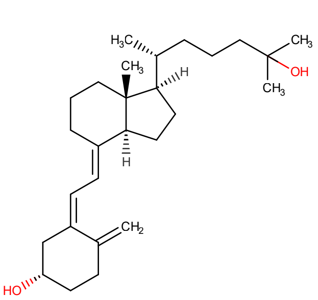

ERGOCALCIFEROL

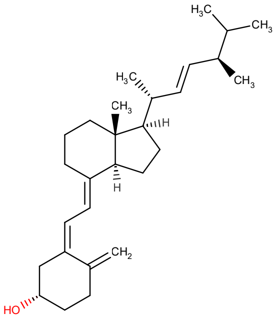

BECOCALCIDIOL

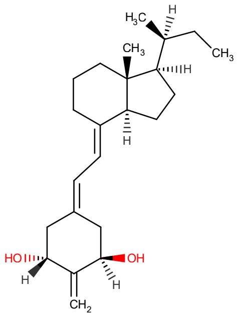

PEFCALCITOL

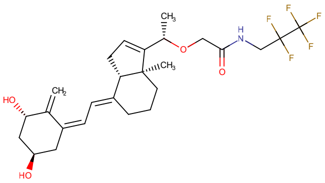

INECALCITOL

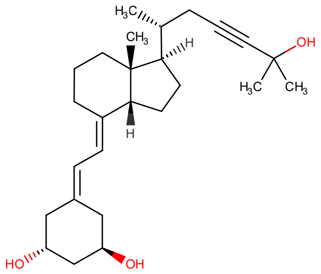

CALCITRIOL

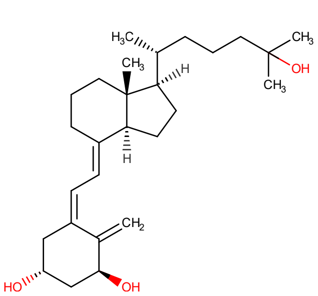

ELOCALCITOL

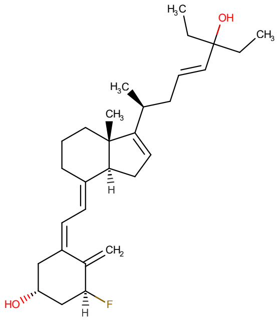

CALCIPOTRIENE

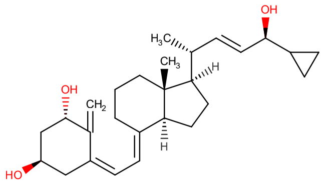

HEXACHLOROPHENE

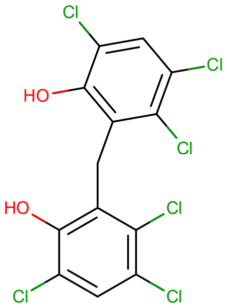

DACTINOMYCIN

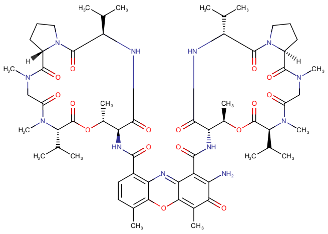

MICONAZOLE

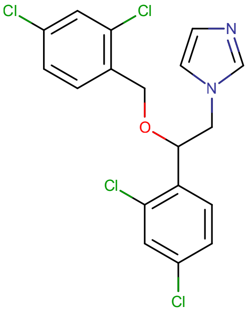

GOSSYPOL

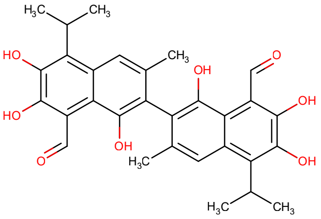

CEFOTAXIME

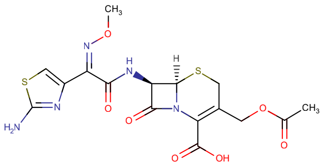

CAFFEINE

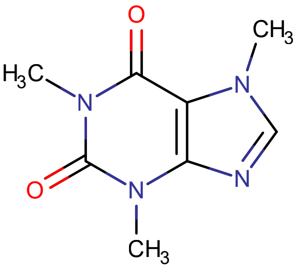

RIBAVIRIN

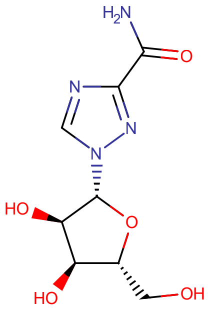

NISOLDIPINE

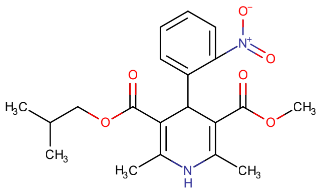

DIPHENADIONE

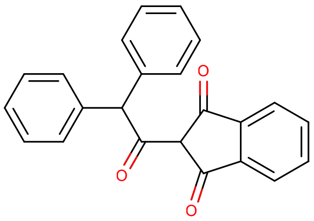

DIPYRITHIONE

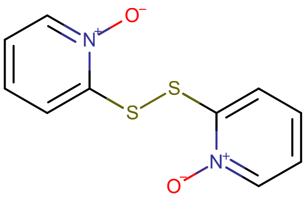

NIFEDIPINE

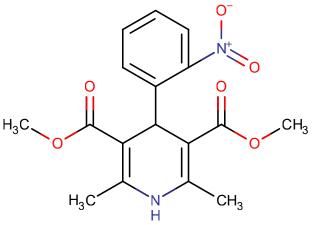

APOMORPHINE

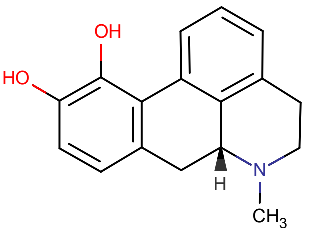

INAMRINONE

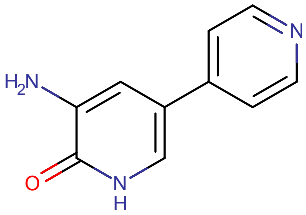

Supplement: Supplementary Figure 6C [file med-2022-0476-Fig-S6C.pdf]
