# Supplementary material for: Epithelial-mesenchymal transition-related genes in coronary artery disease
Source: Open Med (Wars). 2022 Apr 22;17(1):781–800. doi: 10.1515/med-2022-0476 (PMC9034345; doi:10.1515/med-2022-0476)

ETHACRYNIC ACID

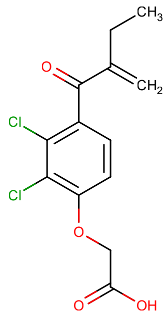

CYCLOSPORINE

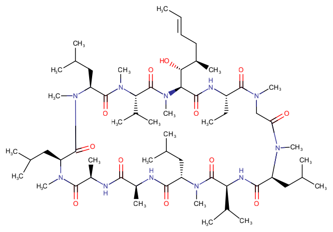

DEFERASIROX

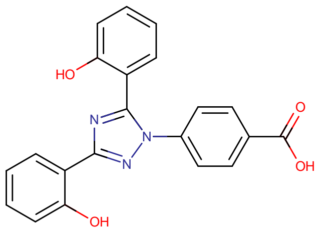

2-MERCAPTOBENZOTHIAZOLE

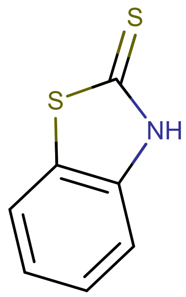

CYSTEAMINE HYDROCHLORIDE

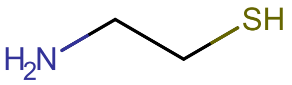

ISOLIQUIRITIGENIN

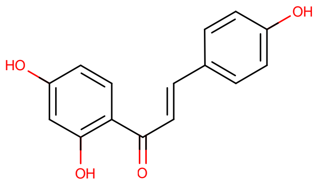

EPINEPHRINE

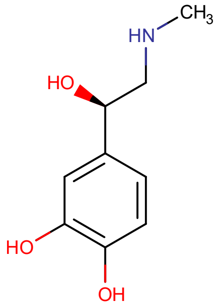

ETHACRIDINE LACTATE

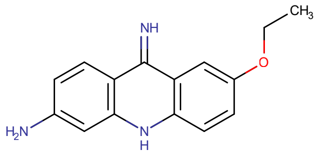

DISULFIRAM

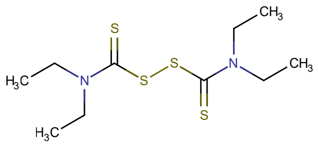

CEFACLOR

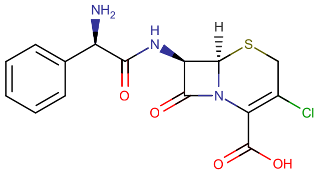

OXYPHENBUTAZONE

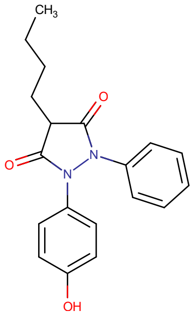

RABEPRAZOLE SODIUM

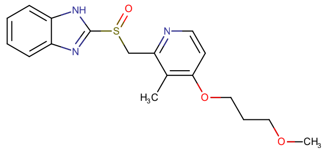

DOCONEXENT

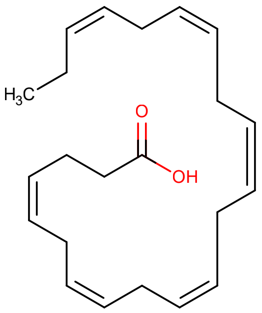

ICOSAPENT

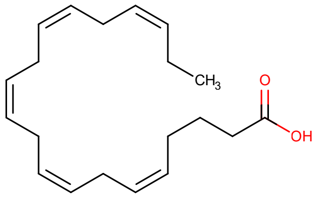

TIOXOLONE

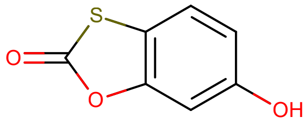

MIDAZOLAM

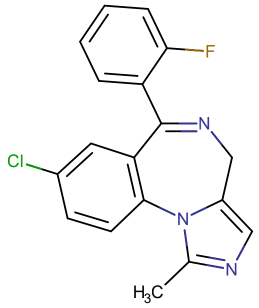

PYRITHIONE

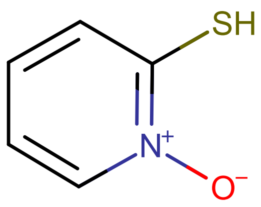

DICLAZURIL

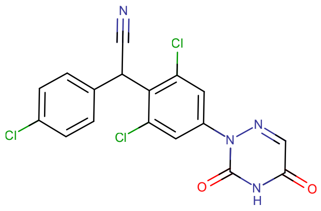

NITAZOXANIDE

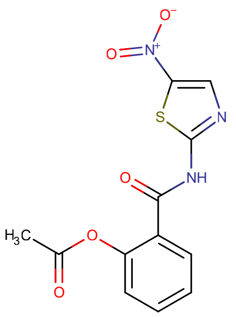

ETHACRIDINE

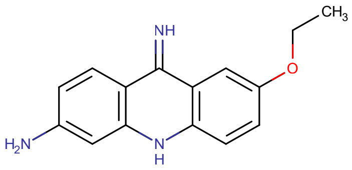

LOFEPRAMINE

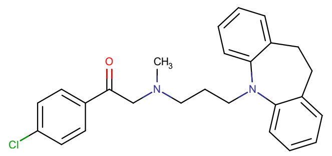

Z-PRO-PROLINAL

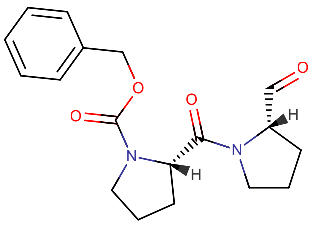

CRYPTOTANSHINONE

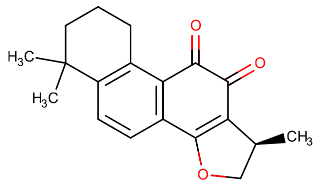

CHINIOFON

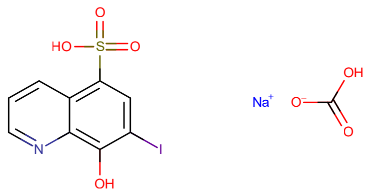

Supplement: Supplementary Figure 6D [file med-2022-0476-Fig-S6D.pdf]
