# Supplementary material for: Epithelial-mesenchymal transition-related genes in coronary artery disease
Source: Open Med (Wars). 2022 Apr 22;17(1):781–800. doi: 10.1515/med-2022-0476 (PMC9034345; doi:10.1515/med-2022-0476)

TESTOSTERONE

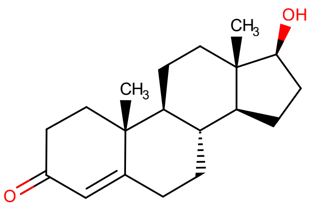

CAMBINOL

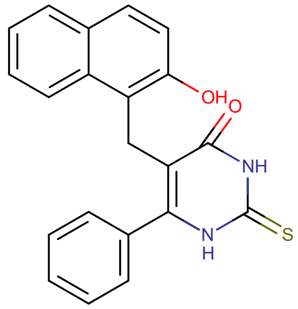

HYDROCORTISONE

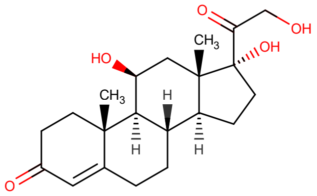

ISOTRETINOIN

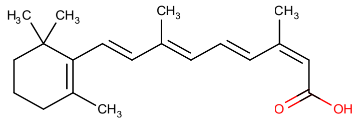

FEPRAZONE

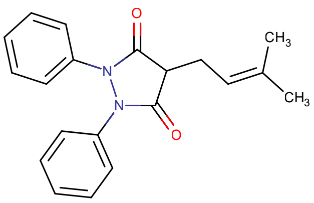

RABEPRAZOLE

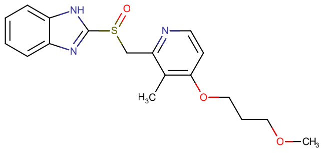

EPALRESTAT

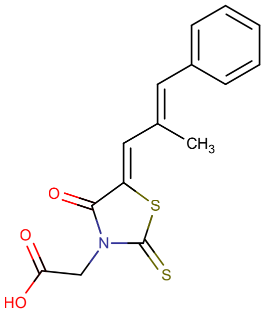

STAUROSPORINE

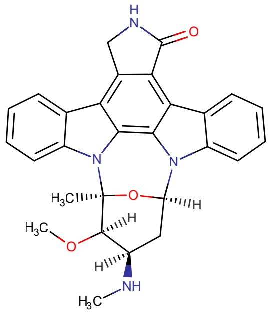

CANDESARTAN CILEXETIL

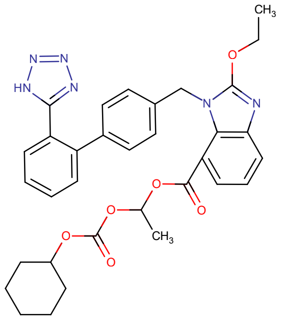

WORTMANNIN

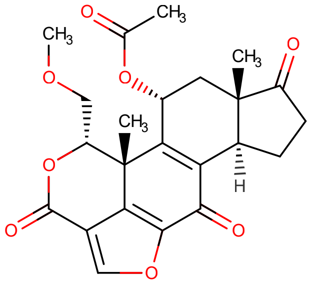

CEFONICID

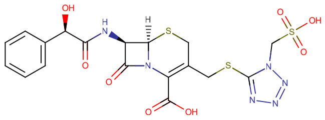

DYCLONINE HYDROCHLORIDE

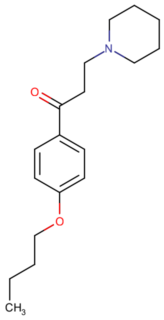

MITOXANTRONE HYDROCHLORIDE

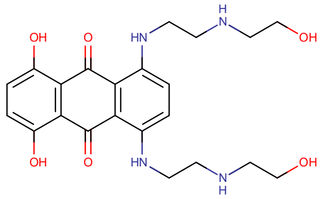

ALENDRONIC ACID

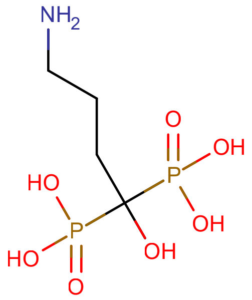

BENSERAZIDE HYDROCHLORIDE

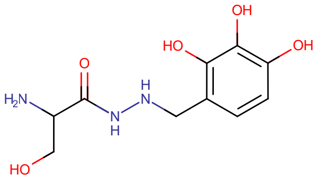

CYSTEINE

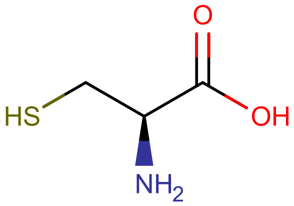

GENTIAN VIOLET

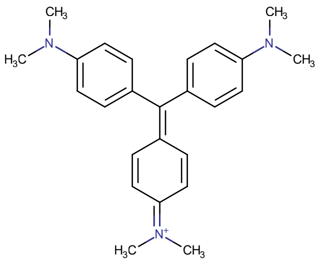

ESTRAMUSTINE

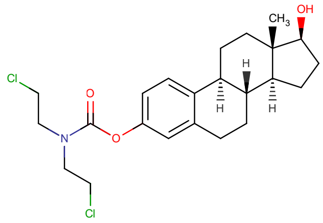

CASODEX

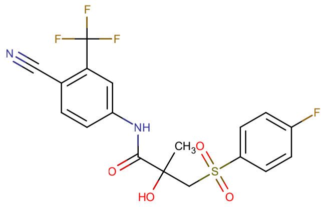

PYRANTEL PAMOATE

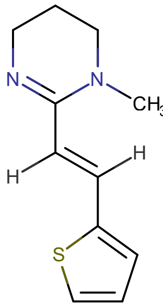

ACITRETIN

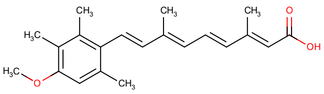

LAPACHONE

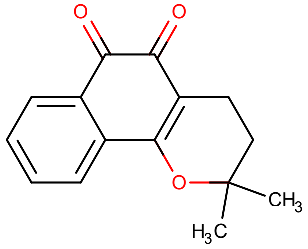

PHOSMET

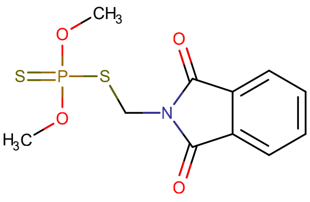

RETINYL ACETATE

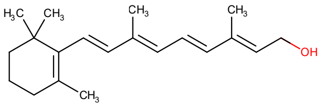

Supplement: Supplementary Figure 6E [file med-2022-0476-Fig-S6E.pdf]
