# Supplementary material for: Epithelial-mesenchymal transition-related genes in coronary artery disease
Source: Open Med (Wars). 2022 Apr 22;17(1):781–800. doi: 10.1515/med-2022-0476 (PMC9034345; doi:10.1515/med-2022-0476)

RALOXIFENE

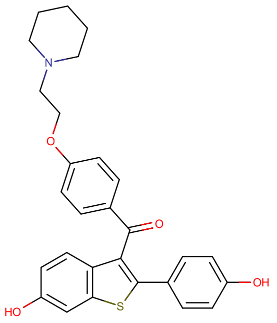

LANSOPRAZOLE

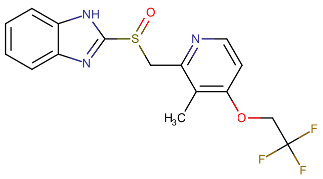

DYCLONINE

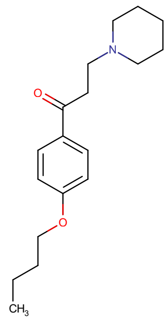

LINOLENIC ACID

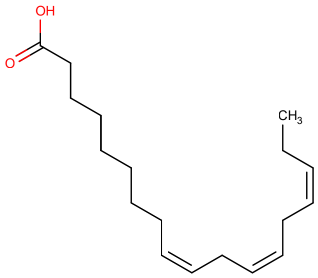

SULFOBROMOPHTHALEIN

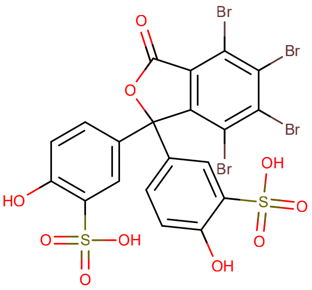

CATECHIN

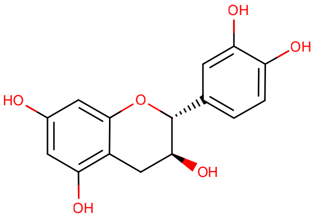

PROTOPORPHYRIN

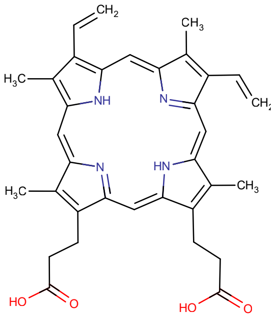

SOFALCONE

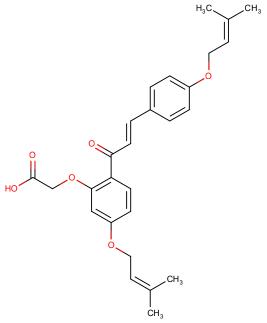

OMEPRAZOLE

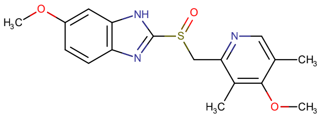

SODIUM LAURYL SULFATE

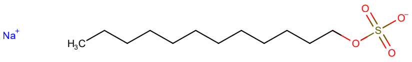

SULFINPYRAZONE

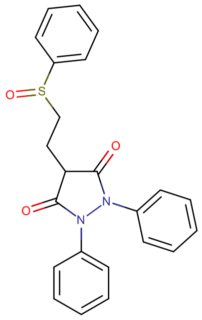

ANETHOLTRITHION

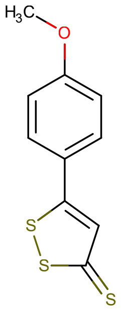

ARACHIDONIC ACID

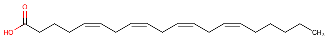

METHYSERGIDE

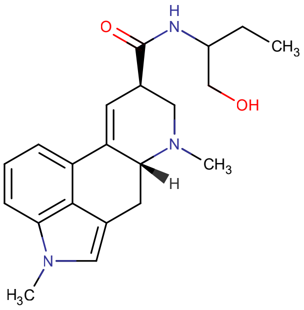

DESERPIDINE

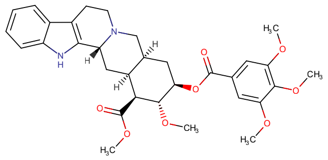

THIOTEPA

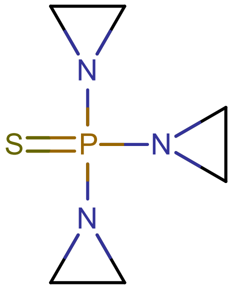

CEFPIROME

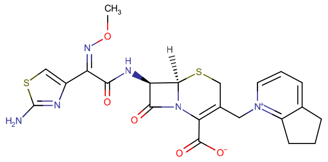

BOCEPREVIR

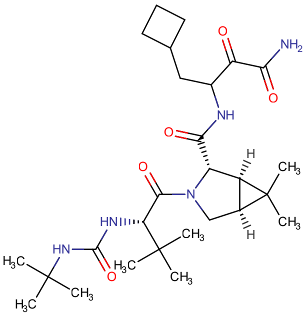

BITHIONOL

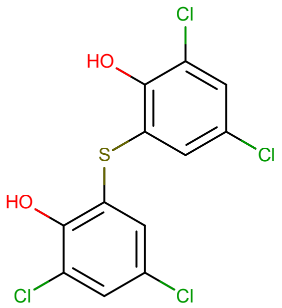

GAMOLENIC ACID

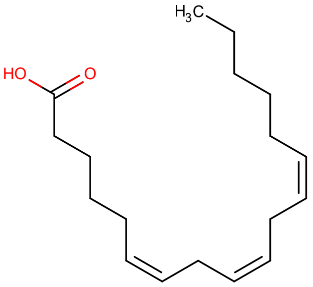

IODOQUINOL

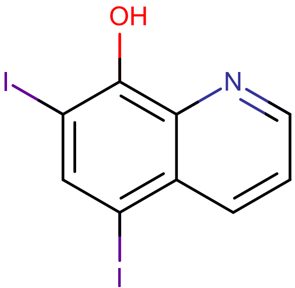

FLUPIRTINE

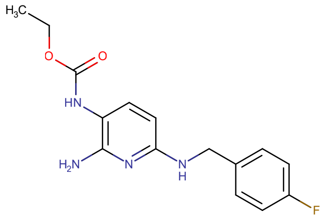

NIFEKALANT

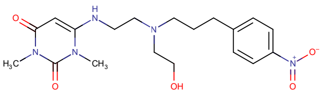

IODOFORM

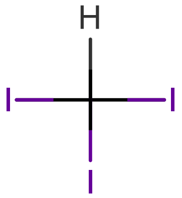

Supplement: Supplementary Figure 6F [file med-2022-0476-Fig-S6F.pdf]
