# Supplementary material for: Epithelial-mesenchymal transition-related genes in coronary artery disease
Source: Open Med (Wars). 2022 Apr 22;17(1):781–800. doi: 10.1515/med-2022-0476 (PMC9034345; doi:10.1515/med-2022-0476)

DEXAMETHASONE

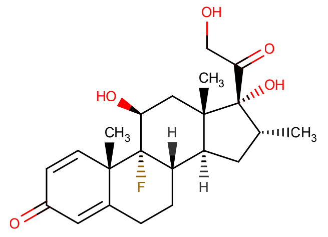

LOVASTATIN

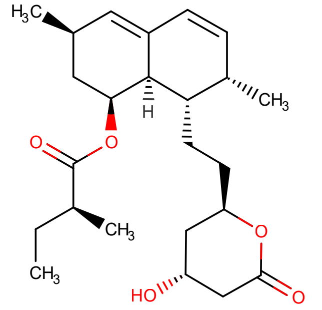

THIRAM

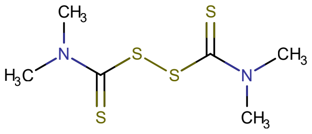

MEDROXYPROGESTERONE ACETATE

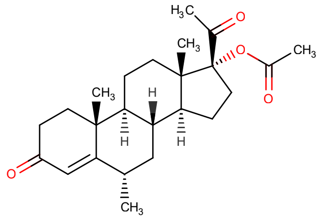

ISOETHARINE

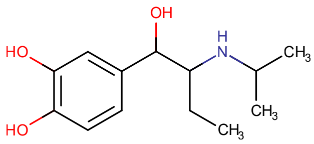

CARBARIL

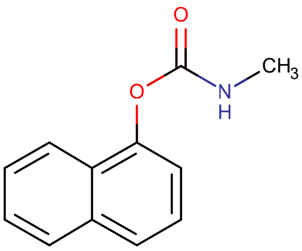

RITANSERIN

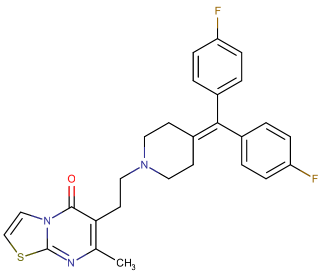

PHANQUONE

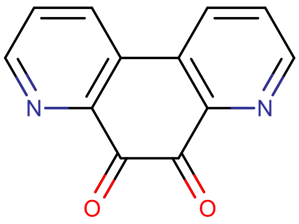

KETOCONAZOLE

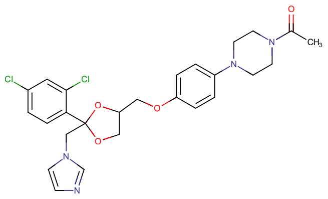

MERSALYL

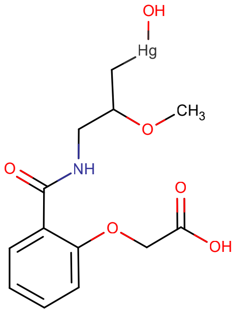

CAMPTOTHECIN

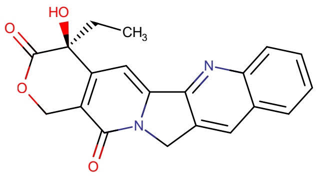

SURAMIN

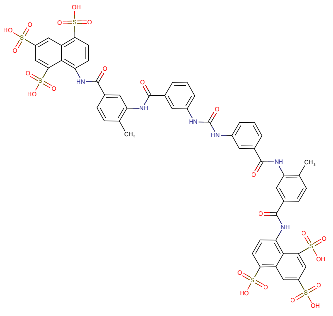

BENZBROMARONE

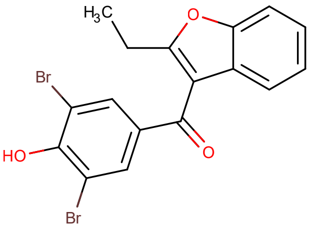

HALOPROGIN

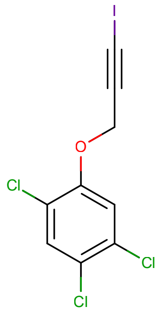

ELDECALCITOL

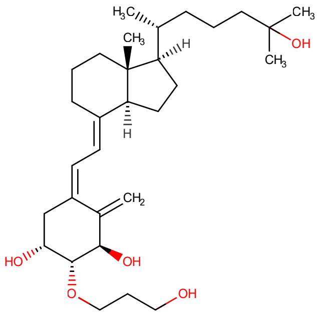

PYRITHIONE ZINC

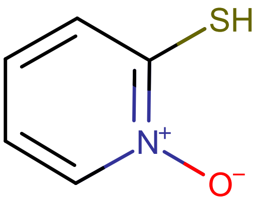

WARFARIN

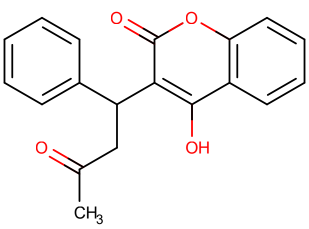

DAUNORUBICIN HYDROCHLORIDE

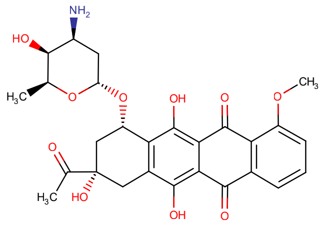

AMOXAPINE

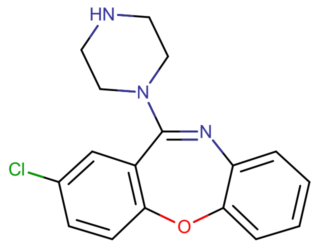

DOCUSATE

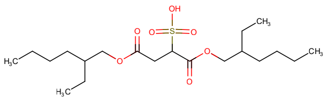

TINORIDINE

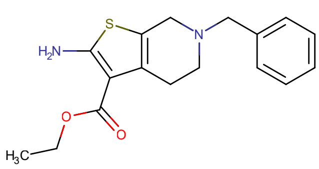

TOLFENAMIC ACID

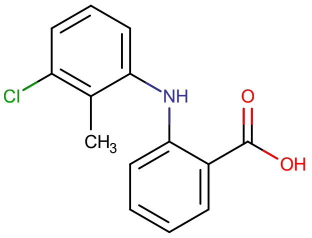

AMBAZONE

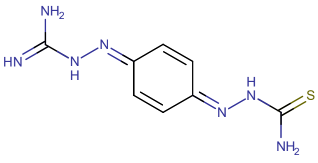

LINTITRIPT

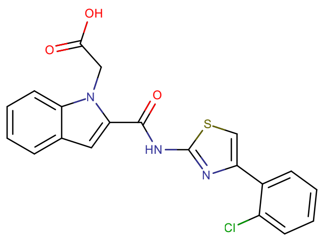

Supplement: Supplementary Figure 6G [file med-2022-0476-Fig-S6G.pdf]
