# Supplementary material for: Epithelial-mesenchymal transition-related genes in coronary artery disease
Source: Open Med (Wars). 2022 Apr 22;17(1):781–800. doi: 10.1515/med-2022-0476 (PMC9034345; doi:10.1515/med-2022-0476)

ELLAGIC ACID

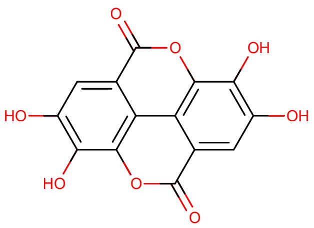

LAWSONE

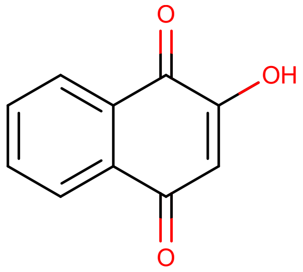

MENADIONE

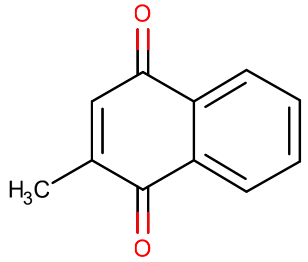

TRIMETREXATE

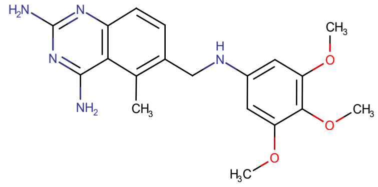

MALATHION

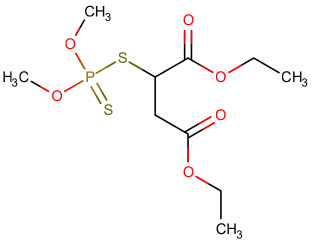

ESTROPIPATE

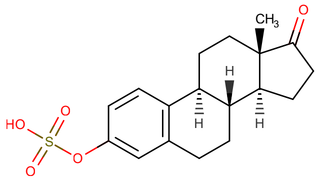

METHYLERGONOVINE

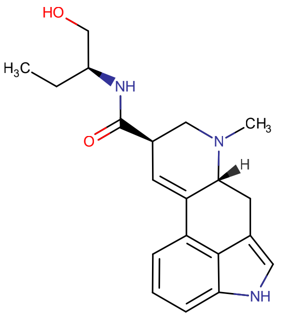

BRONOPOL

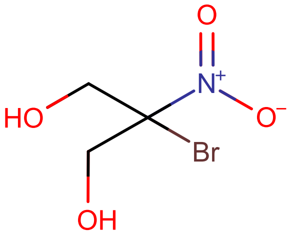

PATULIN

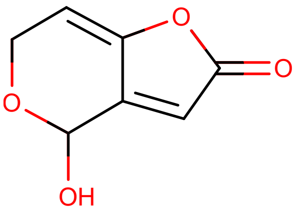

THIMEROSAL

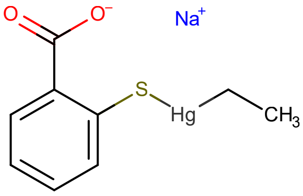

PANTOPRAZOLE

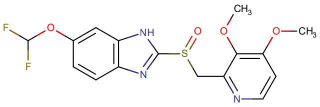

ZOMEPIRAC SODIUM

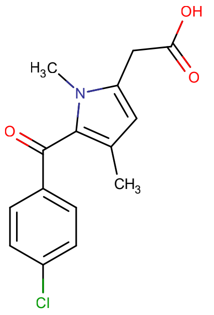

METHOTREXATE

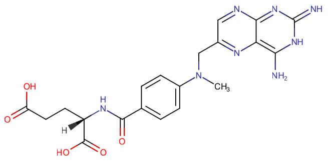

IDEBENONE

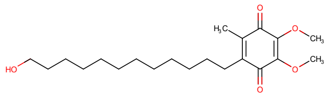

DIXANTHOGEN

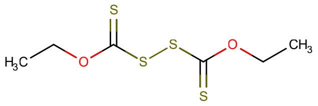

ACRIFLAVINE

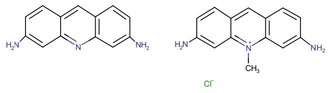

OXYTETRACYCLINE HYDROCHLORIDE

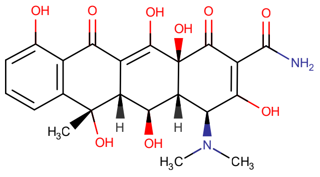

BENZIODARONE

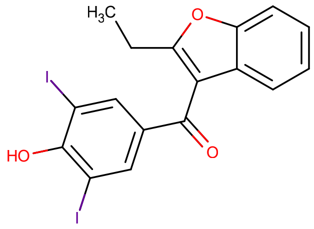

BITHIONOLATE SODIUM

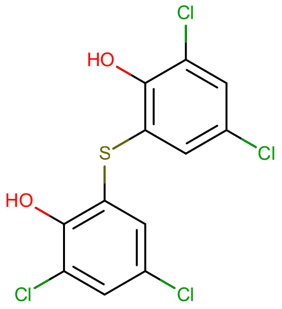

ALITRETINOIN

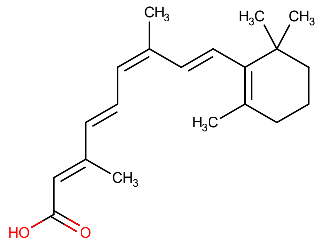

EBSELEN

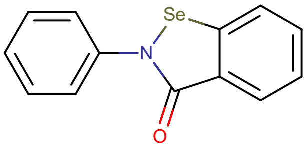

ISOPROTERENOL

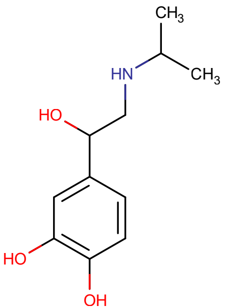

EDARAVONE

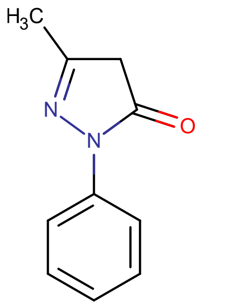

MELENGESTROL ACETATE

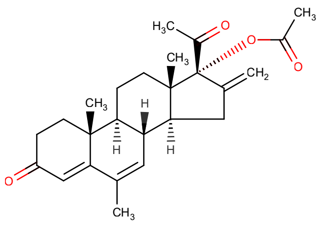

Supplement: Supplementary Figure 6H [file med-2022-0476-Fig-S6H.pdf]
