# Supplementary material for: Epithelial-mesenchymal transition-related genes in coronary artery disease
Source: Open Med (Wars). 2022 Apr 22;17(1):781–800. doi: 10.1515/med-2022-0476 (PMC9034345; doi:10.1515/med-2022-0476)

BRIMONIDINE

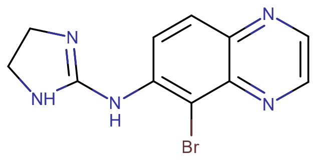

MERCURIC CHLORIDE

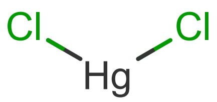

NITROXOLINE

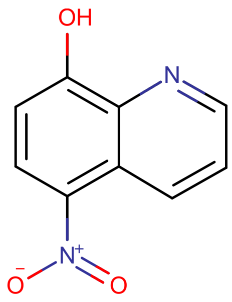

URACIL MUSTARD

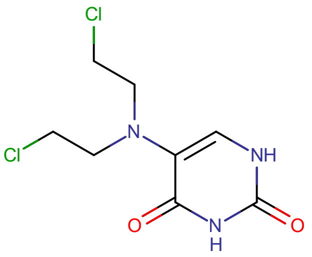

ROSE BENGAL

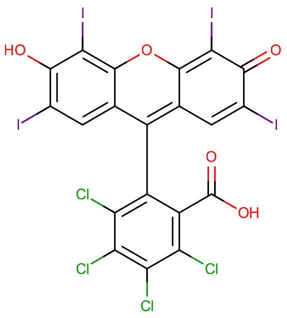

4-AMINOPHENOL

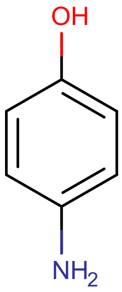

CURCUMIN

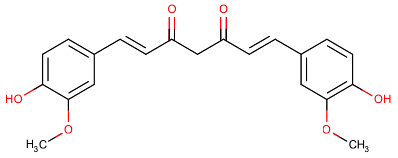

BENSERAZIDE

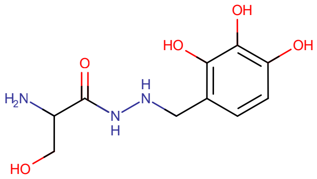

NOREPINEPHRINE

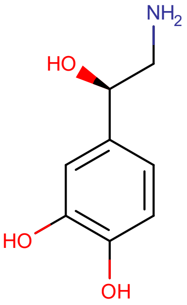

BROMOCRIPTINE

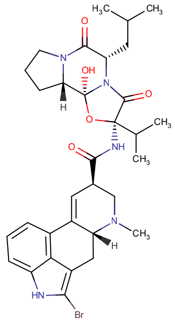

IRINOTECAN

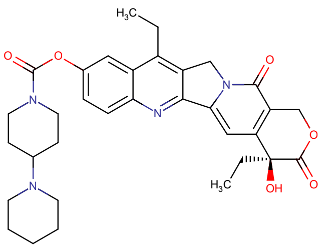

GATIFLOXACIN

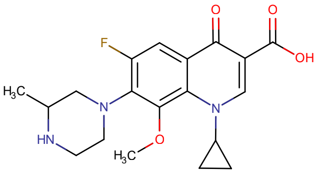

OXCARBAZEPINE

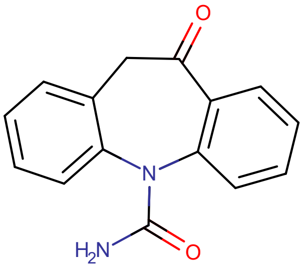

ECONAZOLE NITRATE

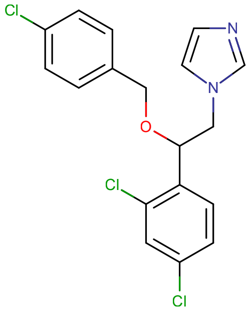

CALCIUM

Ca

NORDIHYDROGUAIARETIC ACID

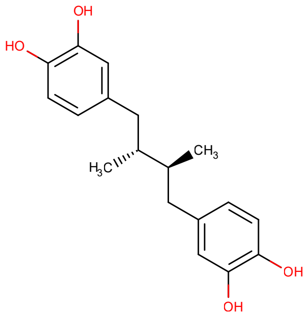

TEMEFOS

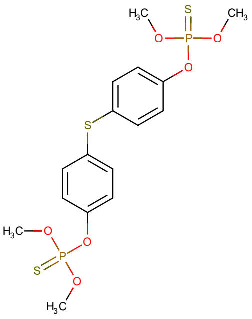

RITONAVIR

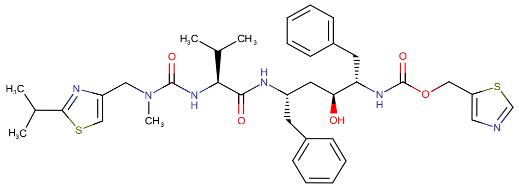

MESALAMINE

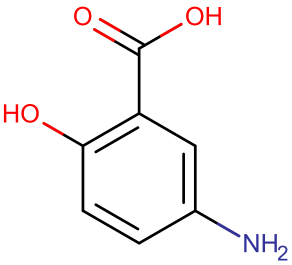

Supplement: Supplementary Figure 6I [file med-2022-0476-Fig-S6I.pdf]
