# Supplementary material for: Epithelial-mesenchymal transition-related genes in coronary artery disease
Source: Open Med (Wars). 2022 Apr 22;17(1):781–800. doi: 10.1515/med-2022-0476 (PMC9034345; doi:10.1515/med-2022-0476)
Supplement: Supplementary Figure 3 [file med-2022-0476-Fig-S3.pdf]

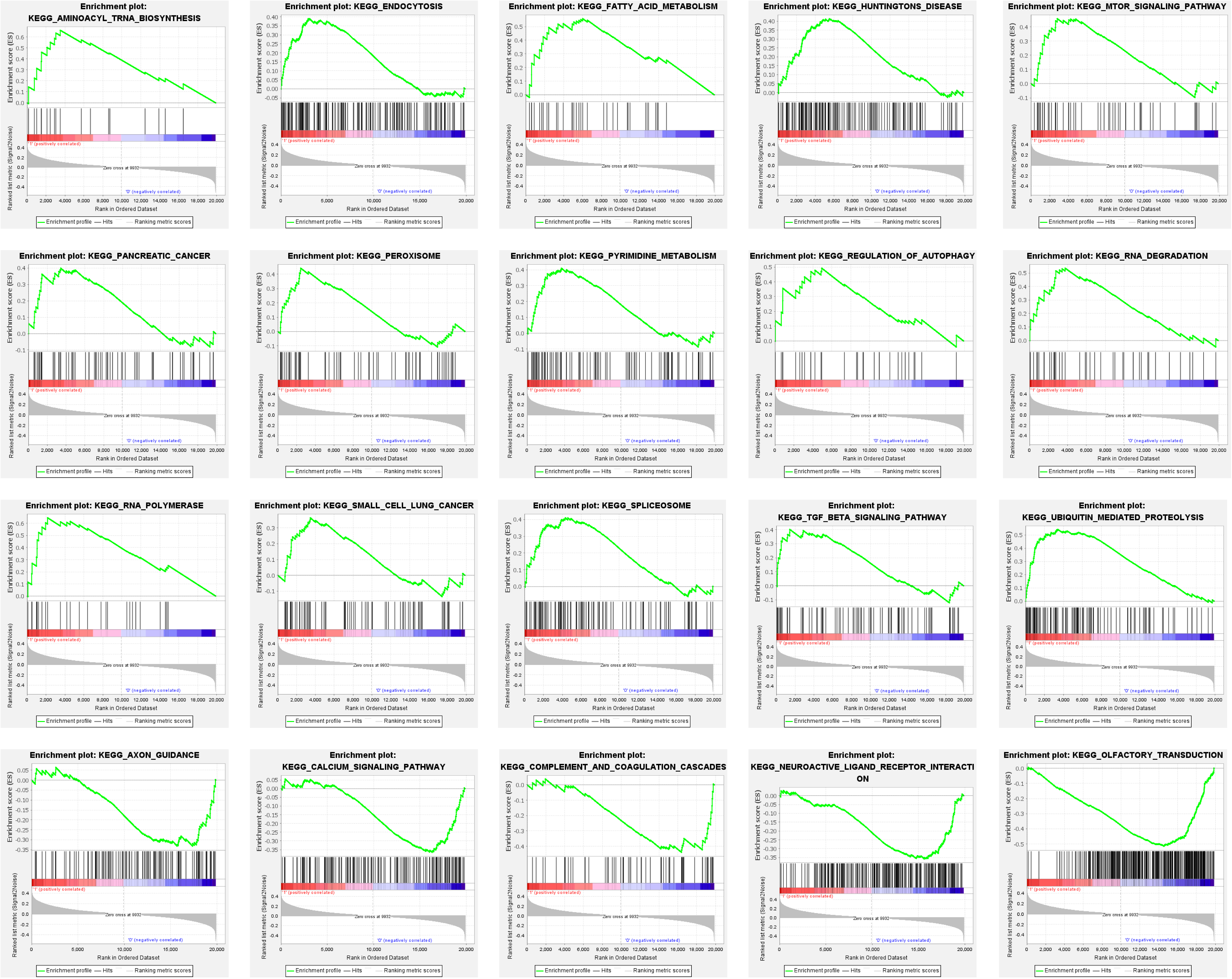
Enrichment plot: KEGG_AMINOACYL_TRNA_BIOSYNTHESIS
Enrichment plot: KEGG_ENDOCYTOSIS
Enrichment plot: KEGG_FATTY_ACID_METABOLISM
Enrichment plot: KEGG_HUNTINGTONS_DISEASE
Enrichment plot: KEGG_MTOR_SIGNALING_PATHWAY
Enrichment plot: KEGG_PANCREATIC_CANCER
Enrichment plot: KEGG_PEROXISOME
Enrichment plot: KEGG_PYRIMIDINE_METABOLISM
Enrichment plot: KEGG_REGULATION_OF_AUTOPHAGY
Enrichment plot: KEGG_RNA_DEGRADATION
Enrichment plot: KEGG_RNA_POLYMERASE
Enrichment plot: KEGG_SMALL_CELL_LUNG_CANCER
Enrichment plot: KEGG_SPLICEOSOME
Enrichment plot: KEGG_TGF_BETA_SIGNALING_PATHWAY
Enrichment plot: KEGG_UBIQUITIN_MEDIATED_PROTEOLYSIS
Enrichment plot: KEGG_AXON_GUIDANCE
Enrichment plot: KEGG_CALCIUM_SIGNALING_PATHWAY
Enrichment plot: KEGG_COMPLEMENT_AND_COAGULATION_CASCADES
Enrichment plot: KEGG_NEUROACTIVE_LIGAND_RECEPTOR_INTERACTION
Enrichment plot: KEGG_OLFACTORY_TRANSDUCTION
Enrichment score (ES)
Ranked list metric (Signal2Noise)
'1' (positively correlated)
Zero cross at 9932
'0' (negatively correlated)
Rank in Ordered Dataset
Enrichment profile — Hits — Ranking metric scores
